# Common variants in toll-like receptor family genes and risk of gastric cancer: a systematic review and meta-analysis

**DOI:** 10.3389/fgene.2023.1280051

**Published:** 2023-11-28

**Authors:** Ayoub Al Othaim, Sulieman Ibraheem Shelash Al-Hawary, Hashem O. Alsaab, Sami G. Almalki, Mazin A. A. Najm, Ahmed Hjazi, Ali Alsalamy, Abbas Firras Almulla, Hamzeh Alizadeh

**Affiliations:** ^1^ Department of Medical Laboratories, College of Applied Medical Sciences, Majmaah University, Al-Majmaah, Saudi Arabia; ^2^ Department of Business Administration, Business School, Al al-Bayt University, Mafraq, Jordan; ^3^ Department of Pharmaceutics and Pharmaceutical Technology, Taif University, Taif, Saudi Arabia; ^4^ Department of Medical Laboratory Sciences, College of Applied Medical Sciences, Majmaah University, Majmaah, Saudi Arabia; ^5^ Pharmaceutical Chemistry Department, College of Pharmacy, Al-Ayen University, Thi-Qar, Iraq; ^6^ Department of Medical Laboratory Sciences, College of Applied Medical Sciences, Prince Sattam Bin Abdulaziz University, Al-Kharj, Saudi Arabia; ^7^ College of Medical Technology, Imam Ja’afar Al‐Sadiq University, Al‐Muthanna, Iraq; ^8^ Medical Laboratory Technology Department, College of Medical Technology, The Islamic University, Najaf, Iraq; ^9^ Genetics Research Center, Department of Genetics and Breeding, The University of Guilan, Rasht, Iran

**Keywords:** gastric cancer, toll-like receptors, polymorphisms, meta-analysis, TLR-4

## Abstract

**Background:** An increasing number of studies have suggested the relationship between single-nucleotide polymorphisms (SNPs) in toll-like receptor (TLR) genes and gastric cancer (GC) susceptibility; however, the available evidence is contradictory. This meta-analysis aimed to comprehensively evaluate whether the SNPs within the TLR family are related to GC development.

**Methods:** PubMed, Scopus, and China National Knowledge Infrastructure (CNKI) were systematically searched up to May 2023 to obtain the pertinent publications. Pooled odds ratios (ORs) with 95% confidence intervals (CIs) were applied to examine the associations using the random-effects model.

**Results:** A total of 45 studies with 25,831 participants (cases: 11,308; controls: 14,523) examining the relation of 18 different SNPs in the TLR family to GC were analyzed. Variations in TLR-4 rs4986790, TLR-4 rs4986791, TLR-5 rs5744174, and TLR-9 rs187084 were significantly associated with increased risk of GC in different genetic models. No significant association was detected for TLR-2-196 to -174de (Delta22), TLR-2 rs3804100, TLR-4 rs11536889, TLR-4 rs11536878, TLR-4 rs2770150, TLR-4 rs10116253, TLR-4 rs1927911, TLR-4 rs10983755, TLR-4 rs10759932, TLR-4 rs1927914, and TLR-10 rs10004195.

**Conclusion:** These findings indicate that variations in TLR-4, TLR-5, and TLR-9 genes were found to be potential risk factors for GC.

## Introduction

Gastric cancer (GC) is the fifth most prevalent kind of malignancy and the third main cause of cancer-related death globally (Castano-Rodriguez et al., 2014), accounting for 8.8% of total cancer-associated mortalities, with 5-year survival rates less than 30% (Castaño-Rodríguez et al., 2014). GC is a complex disease with a multifactorial etiology that includes the combined impacts of lifestyle, bacteria, and host and environmental factors (Gao et al., 2020). However, *Helicobacter pylori* infection is an established risk factor for GC, but only 0.1%–4% of infected people develop GC, highlighting the etiological involvement of environmental predisposing factors and host genetics in GC development (Companioni et al., 2014).

Changes in the host immune elements, such as toll-like receptors (TLRs), may affect the development of GC via their crucial involvement in triggering adaptive and innate immune responses (Sultan et al., 2022). Ten TLRs have been identified on the surfaces of immune cells as well as gastric epithelial cells in humans (Eed et al., 2020). TLRs deliver the first line of host defense against damaging pathogens by recognizing pathogen-associated molecular patterns (PAMPs) expressed by the majority of microorganisms, such as unmethylated CpG motifs (TLR-9), flagella (TLR-5), lipopolysaccharides (LPSs) and lipoproteins (TLR-1,-2,-4,-5, and -6), peptidoglycan (TLR-2), and microbial nucleic acids (TLR-3,-7,-8, and -9) (Castano-Rodriguez et al., 2014; Li et al., 2021). Moreover, TLR-10 expressed by the gastric epithelial cells plays a key role in the identification of multiple patterns of *H. pylori* LPSs (Li et al., 2021). The contribution of TLRs in gastric tumorigenesis has been suggested by previous studies (de Oliveira and Silva, 2012; Castaño-Rodríguez et al., 2014). It has been detected that TLR-3 and TLR-4 are highly expressed in cancer cells (Li et al., 2021). Evidence has also shown that the overexpression of TLR-5 is associated with increased GC cell proliferation and may act as a biomarker for GC (Song et al., 2011; Kasurinen et al., 2019). Furthermore, TLR-7 expression has been negatively linked to the viability of GC cells (Jiang et al., 2016), and the TLR-7 agonist has been examined as a possible vaccine formulation in GC immunotherapy (Wang X-D. et al., 2015). TLR-9 is also expressed aberrantly in GC, and *H. pylori* DNA could trigger TLR-9-mediated GC cell invasion (Gao et al., 2020). Single-nucleotide polymorphisms (SNPs) within TLR genes may lead to a change in their expression along with dysregulation of their signaling pathways, resulting in disturbed secretion of inflammatory mediators and enabling *H. pylori* to cause persistent infection and affecting gastric immunopathology and GC susceptibility (Eed et al., 2020; Sultan et al., 2022).

In recent studies, polymorphisms in TLR genes have been suggested to be associated with the risk of GC (de Oliveira and Silva, 2012; Companioni et al., 2014; Dargiene et al., 2018). However, the results have been inconsistent among various ethnic populations, and the small sample sizes of individual studies limit their statistical power to detect associations. This systematic review and meta-analysis was performed to comprehensively and quantitatively evaluate the relationship between TLR gene polymorphisms and the risk of GC.

## Materials and methods

This meta-analysis was performed according to the PRISMA protocol for systematic review and meta-analysis (Moher et al., 2015).

### Search strategy

Without language restriction, we systematically searched PubMed, Scopus, and China National Knowledge Infrastructure (CNKI) up to 30 May 2023 to find studies examining the relation of polymorphisms in the TLR family to the risk of GC using the following terms: (((((Toll-like receptors[Title/Abstract]) OR (Toll-like receptor[Title/Abstract])) OR (TLR[Title/Abstract])) AND (((((variant*[Title/Abstract]) OR (Polymorphism*[Title/Abstract])) OR (mutation[Title/Abstract])) OR (allele[Title/Abstract])) OR (genotype[Title/Abstract]))) AND ((stomach[Title/Abstract]) OR (gastric[Title/Abstract]))) AND ((((((tumor[Title/Abstract]) OR (cancer[Title/Abstract])) OR (neoplasm[Title/Abstract])) OR (neoplasia[Title/Abstract])) OR (carcinoma[Title/Abstract])) OR (adenocarcinoma[Title/Abstract])). The lists of references within the reviews and pertinent studies were manually searched for possible additional publications. Initially, all studies obtained through PubMed and Scopus were entered into a citation manager software (EndNote) to screen the identified studies according to the inclusion/exclusion criteria. Then, a complementary systematic search was conducted in CNKI for additional studies. Some authors were experts in multiple languages, including Chinese. Nevertheless, except for one non-English study (Chinese) (Zeng et al., 2011a), all other articles published in non-English languages were excluded from the meta-analysis based on their English titles/abstracts because they had unrelated exposures or outcomes.

### Eligibility criteria

Studies with the following criteria were eligible to be included in the present analysis: 1) investigated the association between gene polymorphisms in TLRs (TLR-1, TLR-2, TLR-3, TLR-4, TLR-5, TLR-6, TLR-7, TLR-8, TLR-9, or TLR-10) as the exposure and GC as the outcome; 2) studies with cohort or case-control design; and 3) provided genotype frequency in both cases and controls. We excluded studies that did not have a control group, studies with irrelevant exposure/outcomes, gene expression papers, letters, conference papers, case reports, book chapters, publications with unextractable data, and studies on precancerous gastric lesions. Moreover, studies were excluded from the meta-analysis if the genotypic frequency of the control group deviated from the Hardy–Weinberg equilibrium (HWE). However, for the studies that investigated several polymorphisms, polymorphisms that were not in HWE were excluded from the analysis, and only polymorphisms that were in HWE were included in the meta-analysis. Two reviewers screened titles/abstracts and full texts of the potentially related studies for eligibility assessment based on the inclusion/exclusion criteria. The disagreements regarding the eligibility of studies were resolved by a group discussion among all authors.

### Data extraction and quality assessment

Two investigators independently extracted the data using a standardized data extraction sheet, and disagreements were resolved by discussion between the two investigators, and if necessary, a third reviewer was consulted. The following data were obtained for each publication: the first author’s name, country, sample size of cases and controls, ethnicity, minor allele frequency (MAF) for the control group, HWE, year of publication, and genotype frequencies in controls and cases. The quality of the included publications was assessed using the Q‐Genie tool. This tool includes 11 questions to be marked on a 7‐point Likert scale for assessing several aspects of the genetic association studies. Overall scores ≤35 indicate poor-quality studies, those >35 and ≤45 indicate moderate-quality studies, and those >45 indicate good-quality studies (Sohani et al., 2015).

### Statistical analysis

The HWE in control groups was tested using the chi-squared test, and *p* < 0.05 indicated significant disequilibrium (Alizadeh et al., 2016). The strength of the relation between the TLR polymorphisms and the odds of GC risk was examined using the pooled odds ratio (OR) with a 95% confidence interval (CI) using the random-effects model (DerSimonian–Laird approach) (DerSimonian and Laird, 1986) under five genetic models, namely, the allelic, recessive, dominant, homozygote contrast, and heterozygote contrast models. The heterogeneity among studies was evaluated by the Q-test and I^2^ statistics; *p*-value <0.1 was considered a remarkable evidence of heterogeneity (Abyadeh et al., 2019; Nazari et al., 2022). Subgroup analyses based on ethnicity, age, and source of controls (community-based or hospital-based) were conducted to assess the possible sources of heterogeneity. Publication bias was assessed by funnel plots and Egger’s test, and *p-*values less than 0.05 were considered statistically significant (Alizadeh et al., 2017). All analyses were conducted using Stata software (version 13.0; Stata Corporation, College Station, TX).

## Results

### Description of studies

**FIGURE 1:**
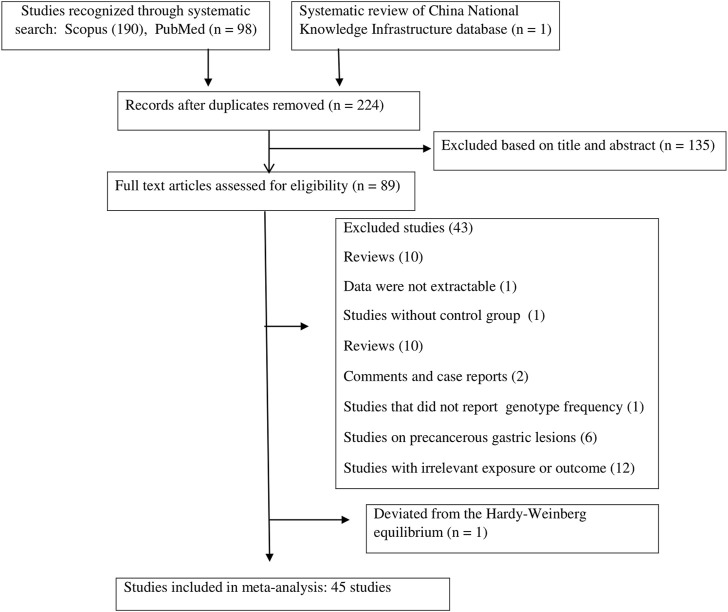
Flow diagram of the study.

A total of 289 publications were obtained through the systematic search of databases. After removing duplicate papers (n = 65) and excluding irrelevant studies based on titles/abstracts (n = 135), a total of 89 studies underwent full-text screening. Of these, 43 publications were excluded according to the inclusion/exclusion criteria. Figure 1 shows the process of study selection and the excluded publications with the specific reasons. For some studies that investigated several SNPs in TLR genes, the genotype frequencies for controls followed the HWE in some SNPs but deviated from the HWE for other SNPs (Schmidt et al., 2011; Ravishankar Ram et al., 2015; Simawaranon et al., 2017; De Re et al., 2019; Susi et al., 2019; Tongtawee et al., 2019). For these studies, only the SNPs that followed the HWE were included in the meta-analysis. Finally, a total of 45 publications (Zeng et al., 2011a; de Oliveira and Silva, 2012; Castano-Rodriguez et al., 2014; Castaño-Rodríguez et al., 2014; Companioni et al., 2014; Dargiene et al., 2018; Eed et al., 2020; Gao et al., 2020; Li et al., 2021; Sultan et al., 2022; Garza-Gonzalez et al., 2007; Hold et al., 2007; Tahara et al., 2007; Santini et al., 2008; Trejo-de la et al., 2008; Hishida et al., 2009; Hold et al., 2009; Hishida et al., 2010; Huang et al., 2010; Rigoli et al., 2010; Zeng et al., 2011b; Kupcinskas et al., 2011; Schmidt et al., 2011; De Oliveira et al., 2013; He et al., 2013; Kim et al., 2013; Wang et al., 2013; Huang et al., 2014; Kutikhin et al., 2014; Li et al., 2014; Qadri et al., 2014; He et al., 2015; Liu et al., 2015; Ravishankar Ram et al., 2015; Stubljar et al., 2015; Wei et al., 2015; Mukherjee et al., 2016; Simawaranon et al., 2017; Xu et al., 2017; He et al., 2018a; De Re et al., 2019; Huang et al., 2019; Susi et al., 2019; Tongtawee et al., 2019; de Matos Lourenço et al., 2020; Gonzalez-Hormazabal et al., 2021), with a total sample size of 25,831 participants (cases: 11,308; controls: 14,523), examining the relation of 18 different SNPs in the TLR family to GC, were eligible to be included in the present meta-analysis. The analyzed studies were published from 2007 to 2022. Moreover, we systematically reviewed the results of the studies on less common SNPs of TLRs and GC that were not eligible for meta-analysis (with only one available effect size) (Zeng et al., 2011b; Kim et al., 2013; Yang et al., 2013; Castano-Rodriguez et al., 2014; Companioni et al., 2014; Liu et al., 2015; De Re et al., 2019; Wang et al., 2019; Gao et al., 2020; Gonzalez-Hormazabal et al., 2021; Li et al., 2021) to have a comprehensive evaluation. Of the included studies in the meta-analysis, 12 studies were on Caucasian populations (Hold et al., 2007; Santini et al., 2008; Hold et al., 2009; Rigoli et al., 2010; Kupcinskas et al., 2011; Companioni et al., 2014; Kutikhin et al., 2014; Stubljar et al., 2015; Dargiene et al., 2018; De Re et al., 2019; Eed et al., 2020; Sultan et al., 2022), seven studies were on Latino populations (Garza-Gonzalez et al., 2007; Trejo-de la et al., 2008; de Oliveira and Silva, 2012; De Oliveira et al., 2013; Susi et al., 2019; de Matos Lourenço et al., 2020; Gonzalez-Hormazabal et al., 2021), and 26 studies were on Asian populations. The mean age of participants ranged from 44.6 ± 15.9 to 66.26 ± 16.32 years. Quality assessment scores of the studies based on the Q-Genie tool are reported in Supplementary Table S1. The overall quality scores of the studies ranged from 23 to 55. Among the studies, 21 studies were rated to be of poor quality, 10 studies were rated to be of moderate quality, and four studies were rated to be of high quality. The genotype frequencies and other characteristics of studies included in the meta-analysis are given in Table 1.

**TABLE 1:** Characteristics of eligible studies considered for the association between toll-like receptor family gene polymorphism and GC risk in the meta-analysis.

				Population characteristics				Genotype frequency									
Authors	Year	Country	Race	Cases	Controls	Source of controls	Case age	Control age	GC cases			Controls				P-HWE	
TLR-1 rs4833095 T>C									TT	TC	CC	TT		TC	CC		
Dargiene	2018	Lithuania, Latvia, and Germany	Caucasian	327	511	Hospital-based	65.49 ± 13.0	47.16 ± 17.3	223	97	7	386		114	11	0.61	
Simawaranon	2017	Thailand	Asian	88	312	based	NR	NR	72	8	8	68		172	72	0.14	
De Re	2019	Italy	Caucasian	114	67	Community-based	61.45 ± 1.04	54.59 ± 1.79	34	65	15	28		30	9	0.83	
TLR-2-196 to -174 del (Delta22)									Ins/ins	Ins/del	Del/del	Ins/ins		Ins/del	Del/del		
Tahara	2007	Japan	Asian	289	146	Community-based	64.9 ± 11.7	36.0 ± 16.11	126	112	51	73		65	8	0.54	
Zeng	2011	China	Asian	248	496	Community-based	57.8	57.8	119	110	19	187		246	63	0.54	
de Oliveira	2012	Brazil	Latino	174	225	Community-based	62.2 ± 12.2	56.5 + 18.1	116	50	8	189		34	2	0.81	
Hishida	2010	Japan	Asian	583	539	Hospital-based	58.8 ± 10.5	58.7 ± 10.6	243	267	73	241		236	62	0.81	
Castano Rodriguez	2014	China	Asian	86	220	Hospital-based	65.3 ± 13.2	54.3 ± 12.8	7	44	35	19		95	106	0.81	
de Oliveira	2013	Brazil	Latino	240	200	Community-based	62.2 ± 12.2	56.5 + 18.1	200	36	4	133		58	9	0.76	
Lourenco	2020	Brazil	Latino	202	381	Hospital-based	66.26 ± 16.32	51.26 ± 16.77	112	79	11	316		60	5	0.60	
Liu	2015	China	Asian	209	94	Community-based	59.2 ± 11.2	55.7 ± 17.3	73	86	50	42		37	15	0.54	
Mukherje	2016	India	Asian	133	266	Community-based	58.7 ± 13.2	58.3 ± 14.0	56	72	5	143		103	20	0.81	
Huang	2019	China	Asian	260	260	Community-based	59.5 ± 12.51	59.0 ± 12.17	105	124	31	132		113	15	0.54	
De Re	2019	Italy	Caucasian	114	67	Community-based	61.45 ± 1.04	54.59 ± 1.79	78	31	5	49		17	1	0.81	
TLR-2 rs3804100 T>C									TT	TC	CC	TT		TC	CC		
Castano Rodriguez	2014	China	Asian	85	212	Hospital-based	65.3 ± 13.2	54.3 ± 12.8	47	34	4	122		76	14	0.65	
Eed	2020	Saudi Arabia	Caucasian	45	80	Hospital-based	45 ± 17.7	42 ± 22.3	29	13	3	60		18	2	0.65	
*Tongtawee*	2018	*Thailand*	Asian	88	312	Hospital-based	44.6 ± 15.9	44.6 ± 15.9	66	22	0	230		70	12	0.09	
TLR-2 rs3804099 T>C									TT	TC	CC	TT		TC	CC		
Gonzalez-Hormazabal	2021	Chile	Latino	299	301	Community-based	25 to 93	18 to 82	32	136	131	41		125	135	0.42	
Lourenco	2020	Brazil	Latino	202	381	Hospital-based	66.26 ± 16.32	51.26 ± 16.77	101	83	18	157		175	49	0.98	
Zeng	2011	China	Asian	248	496	Community-based	58.6	59	132	99	17	216		231	49	0.43	
De Re	2019	Italy	Caucasian	114	67	Community-based	61.45 ± 1.04	54.59 ± 1.79	32	62	20	17		37	13	0.47	
TLR-4 rs1927914 T>C									TT	TC	CC	TT		TC	CC		
Huang	2010	China	Asian	946	987	Community-based	60.63 ± 10.18	60.05 ± 10.36	339	450	157	358		476	153	0.53	
Li	2021	China	Asian	471	471	Community-based	NR	NR	173	233	65	149		241	81	0.79	
TLR-4 rs4986790 (+896 A < G)									AA	AG	GG	AA		AG	GG		
Santini	2008	Italy	Caucasian	171	151	Community-based	60 ± 12	56 ± 16	159	11	1	140		11	0	0.64	
Companioni	2014	Some European countries	Caucasian	361	1,270	Hospital-based	58.4 + 7.93	58.4 + 7.69	316	45	0	1,134		133	3	0.66	
de Oliveira	2012	Brazil	Latino	174	225	Community-based	62.2 ± 12.2	56.5 + 18.1	154	20	0	215		10	0	0.73	
de Oliveira	2013	Brazil	Latino	200	240	Community-based	61.7 ± 12.5	55.5 ± 17.7	174	26	0	224		16	0	0.59	
Garza-Gonzalez	2007	Mexico	Latino	78	189	Hospital-based	58.6	57.1	72	6	0	175		14	0	0.60	
Hold^a^	2007	Poland	Caucasian	312	419	Community-based	NR	NR	258	51	3	387		31	1	0.65	
Hold^b^		United States	Caucasian	184	184		NR	NR	156	28	0	156		28	0	0.26	
Trejo-de la	2008	Mexico	Latino	38	144	Hospital-based	60.9 ± 13.9	47.8 ± 12.2	34	4	0	138		6	0	0.80	
Rigoli	2010	Italy	Caucasian	60	87	Community-based	NR	NR	42	18	0	80		7	0	0.70	
Qadri	2014	India	Asian	130	200	Hospital-based	NR	NR	107	23	0	169		31	0	0.23	
Kutikhin	2014	Russia	Caucasian	57	300	Community-based	58.7 ± 4.1	58.3 ± 3.5	46	11	0	258		39	3	0.27	
Schmidt	2011	China	Asian	60	162	Hospital-based	NR	NR	59	1	0	147		13	2	0.11	
Eed	2020	Saudi Arabia	Caucasian	45	80	Hospital-based	45 ± 17.7	42 ± 22.3	26	10	9	56		21	3	0.57	
Stubljar	2015	Slovenia	Caucasian	32	108	Community-based	52 ± 10	52 ± 10	30	2	0	90		17	1	0.84	
TLR-4 rs4986791 C < T									CC	CT	TT	CC		CT	TT		
Garza-Gonzalez	2007	Mexico	Latino	78	189	Hospital-based	58.6	57.1	77	1	0	179		10	0	0.86	
Santini	2008	Italy	Caucasian	171	151	Community-based	60 ± 12	56 ± 16	155	15	1	147		4	0	0.86	
Trejo-de la	2008	Mexico	Latino	61	202	Hospital-based	60.9 ± 13.9	47.8 ± 12.2	57	4	0	193		9	0	0.86	
Rigoli	2010	Italy	Caucasian	70	87	Community-based	NR	NR	57	13	0	81		6	0	0.86	
de Oliveira	2012	Brazil	Latino	174	225	Community-based	62.2 ± 12.2	56.5 + 18.1	165	9	0	219		6	0	0.86	
de Oliveira	2013	Brazil	Latino	200	240	Community-based	61.7 ± 12.5	55.5 ± 17.7	191	9	0	234		6	0	0.86	
Kutikhin	2014	Russia	Caucasian	66	300	Community-based	58.7 ± 4.1	58.3 ± 3.5	55	11	0	255		45	0	0.58	
Companioni	2014	Some European countries	Caucasian	354	1,263	Hospital-based	58.4 + 7.93	58.4 + 7.69	309	45	0	1,124		134	5	0.86	
Qadri	2014	India	Asian	130	200	Hospital-based	NR	NR	114	16	0	182		18	0	0.86	
Eed	2020	Saudi Arabia	Caucasian	45	80	Hospital-based	45 ± 17.7	42 ± 22.3	29	10	6	63		14	3	0.4	
TLR-4 rs11536889 G>C									GG	GC	CC	GG		GC	CC		
Hishida	2009	Japan	Asian	583	1,592	Hospital-based	58.8 ± 10.5	58.8 ± 10.6	312	222	49	827		635	130	0.94	
Kupcinskas	2011	Germany	Caucasian	113	236	Hospital-based	65.51 ± 13.4	56.9 ± 16.1	90	21	2	190		41	5	0.52	
Companioni	2014	Some European countries	Caucasian	365	1,283	Hospital-based	58.4 + 7.93	58.4 + 7.69	258	98	9	940		308	35	0.52	
He	2013	China	Asian	231	539	Hospital-based	60.03 ± 12.65	48.70 ± 11.64	146	73	12	343		175	21	0.94	
Li	2014	China	Asian	409	409	Hospital-based	59.02 ± 11	53.07 ± 9.84	331	74	4	328		77	4	0.94	
He	2018	China	Asian	479	483	Hospital-based	64.48 ± 11.91	64.73 ± 11.84	303	156	20	293		166	24	0.94	
Castano-Rodriguez	2014	China	Asian	85	212	Hospital-based	65.3 ± 13.2	54.3 ± 12.8	43	33	9	131		74	7	0.75	
Wei	2015	China	Asian	153	100	Community-based	58.2 ± 10.2	55.6 ± 1.3	95	54	4	64		34	2	0.75	
TLR-4 rs10759932 T>C									TT	TC	CC	TT		TC	CC		
He	2018	China	Asian	479	483	Hospital-based	64.48 ± 11.91	64.73 ± 11.84	240	191	48	251		196	36	0.79	
Castano-Rodriguez	2014	China	Asian	85	212	Hospital-based	65.3 ± 13.2	54.3 ± 12.8	54	24	7	115		86	11	0.67	
Kim	2013	Korea	Asian	459	487	Hospital-based	54.8 ± 8.4	54.3 ± 7.4	246	176	37	272		182	33	0.79	
*Tongtawee*	2018	Thailand	Asian	88	312	Hospital-based	44.6 ± 15.9	44.6 ± 15.9	76	0	12	216		90	6	0.67	
Huang	2010	China	Asian	909	1,053	Community-based	60.63 ± 10.18	60.05 ± 10.36	516	316	77	541		432	80	0.79	
Wei	2015	China	Asian	150	100	Community-based	58.2 ± 10.2	55.6 ± 1.3	90	53	7	42		40	18	0.67	
TLR-4 rs1927911 C>T									CC	CT	TT	CC		CT	TT		
He	2018	China	Asian	479	483	Hospital-based	64.48 ± 11.91	64.73 ± 11.84	171	226	82	175		226	82	0.53	
Castano-Rodriguez	2014	China	Asian	77	221	Hospital-based	65.3 ± 13.2	54.3 ± 12.8	42	27	8	80		110	31	0.53	
Huang	2014	China	Asian	217	294	Hospital-based	NR	NR	79	110	28	86		137	71	0.53	
TLR-4 rs10116253 T>C									TT	TC	CC	TT		TC	CC		
Castano-Rodriguez	2014	China	Asian	85	196	Hospital-based	65.3 ± 13.2	54.3 ± 12.8	42	32	11	80		105	28	0.66	
He	2013	China	Asian	230	498	Hospital-based	60.03 ± 12.65	48.70 ± 11.64	85	102	43	199		256	89	0.66	
Huang	2014	China	Asian	217	251	Hospital-based	NR	NR	82	107	28	85		138	71	0.66	
TLR-4 rs2770150 T>C									TT	TC	CC	TT		TC	CC		
Castano-Rodriguez	2014	China	Asian	85	213	Hospital-based	65.3 ± 13.2	54.3 ± 12.8	85	0	0	210		3	0	0.91	
Gonzalez-Hormazabal	2021	Chile	Latino	299	301	Community-based	25 to 93	18–82	23	112	164	20		135	146	0.25	
TLR-4 rs11536878 C>A									CC	CA	AA	CC		CA	AA		
He	2013	China	Asian	233	549	Hospital-based	60.03 ± 12.65	48.70 ± 11.64	183	46	4	434		105	10	0.44	
He	2015	China	Asian	1,262	696	Hospital-based	NR	NR	1,008	25^∑^		558		13^∑^		-	
Li	2014	China	Asian	409	409	Hospital-based	59.02 + 11.08	53.07 + 9.84	331	74	4	328		77	4	0.82	
TLR-4 rs10983755 G>A									GG	GA	AA	GG		GA	AA		
He	2015	China	Asian	1,035	686	Hospital-based	NR	NR	531	50^∑^		355		33^∑^		-	
Kim	2013	Korea	Asian	459	487	Hospital-based	54.8 ± 8.4	54.3 ± 7.4	259	165	35	286		172	29	0.64	
Li	2014	China	Asian	409	409	Hospital-based	59.02 + 11.08	53.07 + 9.84	213	152	44	177		188	44	0.64	
TLR-5 rs5744174 T>C									TT	TC	CC	TT		TC	CC		
Xu	2017	China	Asian	114	67	NR	59.99 ± 10.56	59.35 ± 9.76	794	425	81	858		390	52	0.54	
Zeng	2011	China	Asian	114	67	Hospital-based	57.8	57.8	144	89	15	324		156	16	0.59	
De Re	2019	Italy	Caucasian	114	67	Community-based	61.45 ± 1.04	54.59 ± 1.79	47	44	23	23		37	7	0.48	
TLR-9 rs187084 T>C									TT	TC	CC	TT		TC	CC		
Gao	2020	China	Asian	286	272	Community-based	59.48 ± 11.23	59.10 ± 11.57	96	134	56	93		136	43	0.85	
Sultan	2022	Egypt	Caucasian	106	106	Hospital-based	56.55 ± 8.63	56.55 ± 8.63	22	28	56	40		42	24	0.15	
Liu	2015	China	Asian	209	94	Community-based	59.2 ± 11.2	55.7 ± 17.3	58	111	40	29		55	10	0.15	
Wang	2013	China	Asian	314	314	Community-based	60 ± 12.61	59 ± 12.01	94	164	56	122		148	44	0.93	
Susi	2019	Brazil	Latino	161	200	Hospital-based	63.33 ± 14.45	49.58 ± 21.01	21	101	39	58		106	36	0.60	
Zeng	2011	China	Asian	248	496	Community-based	58.6	59	89	124	35	165		243	88	0.93	
TLR-9 -1237 T>C (rs5743836)									TT	TC	CC	TT		TC	CC		
De Re	2019	Italy	Caucasian	114	67	Community-based	61.45 ± 1.04	54.59 ± 1.79	80	30	4	55		12	0	0.56	
Hold	2009	Poland	Caucasian	326	406	Community-based	NR	NR	261	58	7	316		85	5	0.78	
TLR-10 rs10004195 A < T									AA	AT	TT	AA		AT	TT		
Eed	2020	Saudi Arabia	Caucasian	45	80	Hospital-based	45 ± 17.7	42 ± 22.3	18	13	14	33		30	17	0.052	
Ram	2015	Malaysia	Asian	15	70	Hospital-based	18 to 80	18 to 80	6	5	4	27		24	19	0.06	

P-HWE, *p*-value for Hardy–Weinberg equilibrium.

^∑^ Sum of homozygous and heterozygote variants.

### Systematic review of rare polymorphisms in TLR genes and GC

The characteristics of studies on less common SNPs of TLRs and GC that were not included in the meta-analysis but were included in the systematic review are presented in Supplementary File S1 (1, 3, 4, 7, 25, 39, 42, 49, and 57–59). Castano-Rodriguez et al. (2014) investigated the relation between TLR-4 SNPs (rs10759931, rs11536891, rs11536898, rs2149356, and rs5030728) and GC risk in a Chinese population and found that rs10759931 in TLR-4 could provide protection against GC in the dominant model and heterozygote contrast model, while no association for other SNPs was detected. Companioni et al. (2014) examined the effect of TLR-4 SNPs (rs1329061, rs1329060, rs1329057, and rs10491851) in Caucasian populations and reported that the carriers of the variant allele of the rs10491851 in TLR-4 were significantly at lower risk of GC. In the study by Gonzalez-Hormazabal et al. (2021) in Chile on TLR-4-2 rs7656411, TLR-4 rs1554973, TLR-4 rs7037117, TLR-4 rs913930, and TLR-5 rs75977922, a significantly increased odds of GC was observed for carriers of the variant allele/genotype of TLR-5 rs75977922. No significant association was identified between the TLR-1 rs5743681 in German (Yang et al., 2013), TLR-2 rs1898830 in Korean (Kim et al., 2013), TLR-8 rs3764880 in Italian (De Re et al., 2019), and TLR-9 rs164640 in Chinese (Gao et al., 2020) populations and the risk of GC. In contrast, variations in TLR-3 1377C T (rs3775290) (Liu et al., 2015) and TLR-4 rs1057317 (Trejo-de la et al., 2015; Wang et al., 2019) in Chinese populations were significantly linked to the increased risk of GC among the risk-allele carriers. Li et al. (2021), in 2020, revealed a significant negative relationship between the TLR-4 rs7873784 (dominant model: OR = 0.17 (95% CI: 0.09–0.33) and TLR-4 rs7869402 [dominant model: OR = 0.61 (95% CI: 0.40–0.92)] and GC risk, while no association was reported for TLR-3 rs5743303, TLR-4 rs1927914, TLR-5 rs1640816, and TLR-7 rs3853839 and GC risk. Moreover, in the Chinese population, compared to carriers of the C allele, carriers of the risk allele (T) of TLR-3 1377C < T (rs3775290) showed a 2.05-fold elevated odds of GC, OR = 2.02 (95% CI: 1.41–2.98) (Liu et al., 2015). Zeng et al. (2011b) also investigated the association of two common SNPs in TLR-2 and TLR-5 genes (TLR-5 rs2072493, TLR-5-889T>C, and TLR-2 -688G>T) with GC and reported that these variants are not significant predictors of GC susceptibility. Overall, these findings evidentiate that polymorphisms within the TLR genes may be potential predictors of GC; however, the available data for these SNPs are rare, and further studies are required to assess these relationships.

### Meta-analysis

**FIGURE 2:**
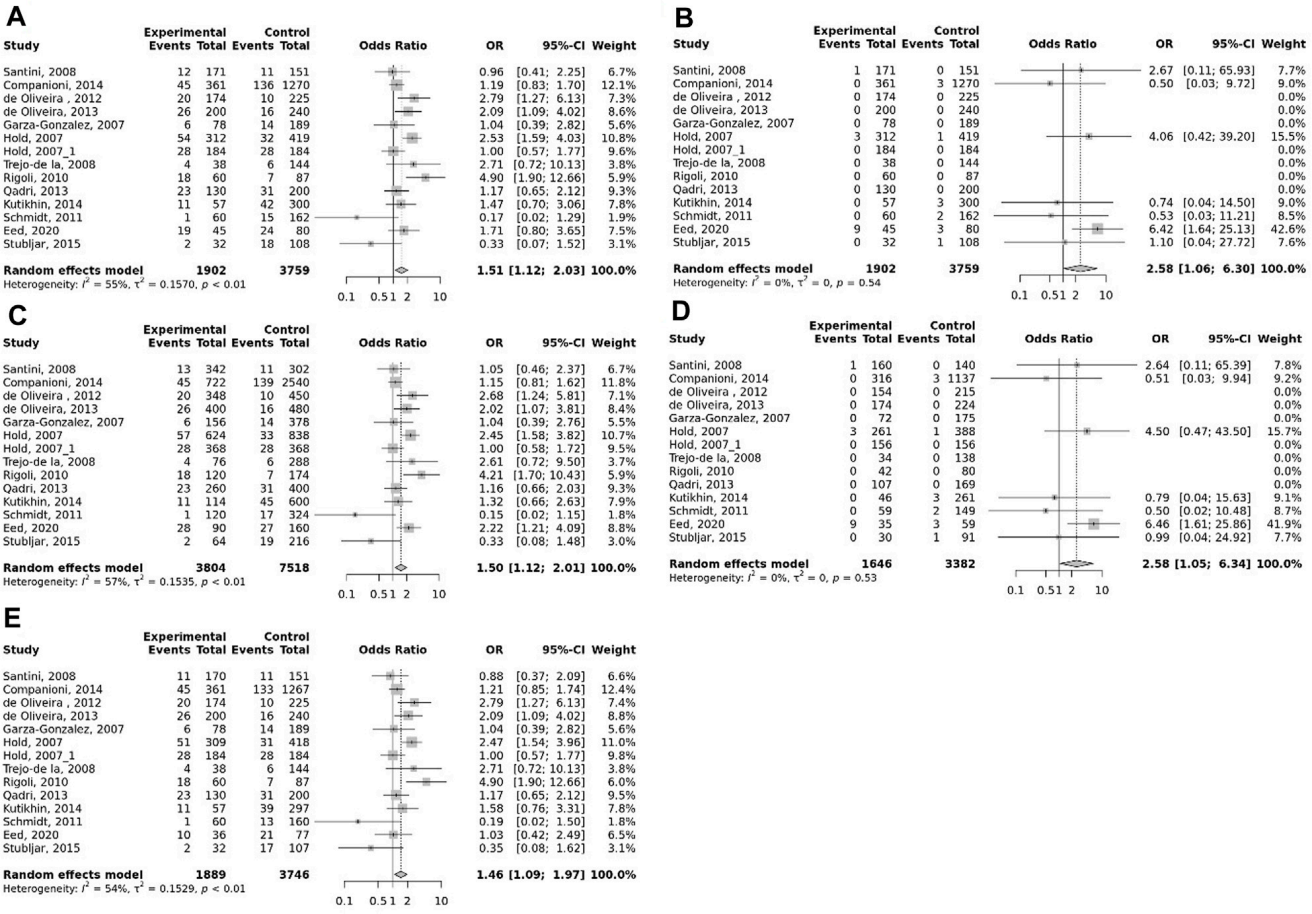
Meta-analysis of the association between TLR-4 rs4986790 (+896 A<G) polymorphism with gastric cancer in dominant **(A)**, recessive **(B)**, allelic **(C)**, homozygote (GG vs. AA) **(D)**, and heterozygote (AG vs. AA) **(E)** models.

**FIGURE 3:**
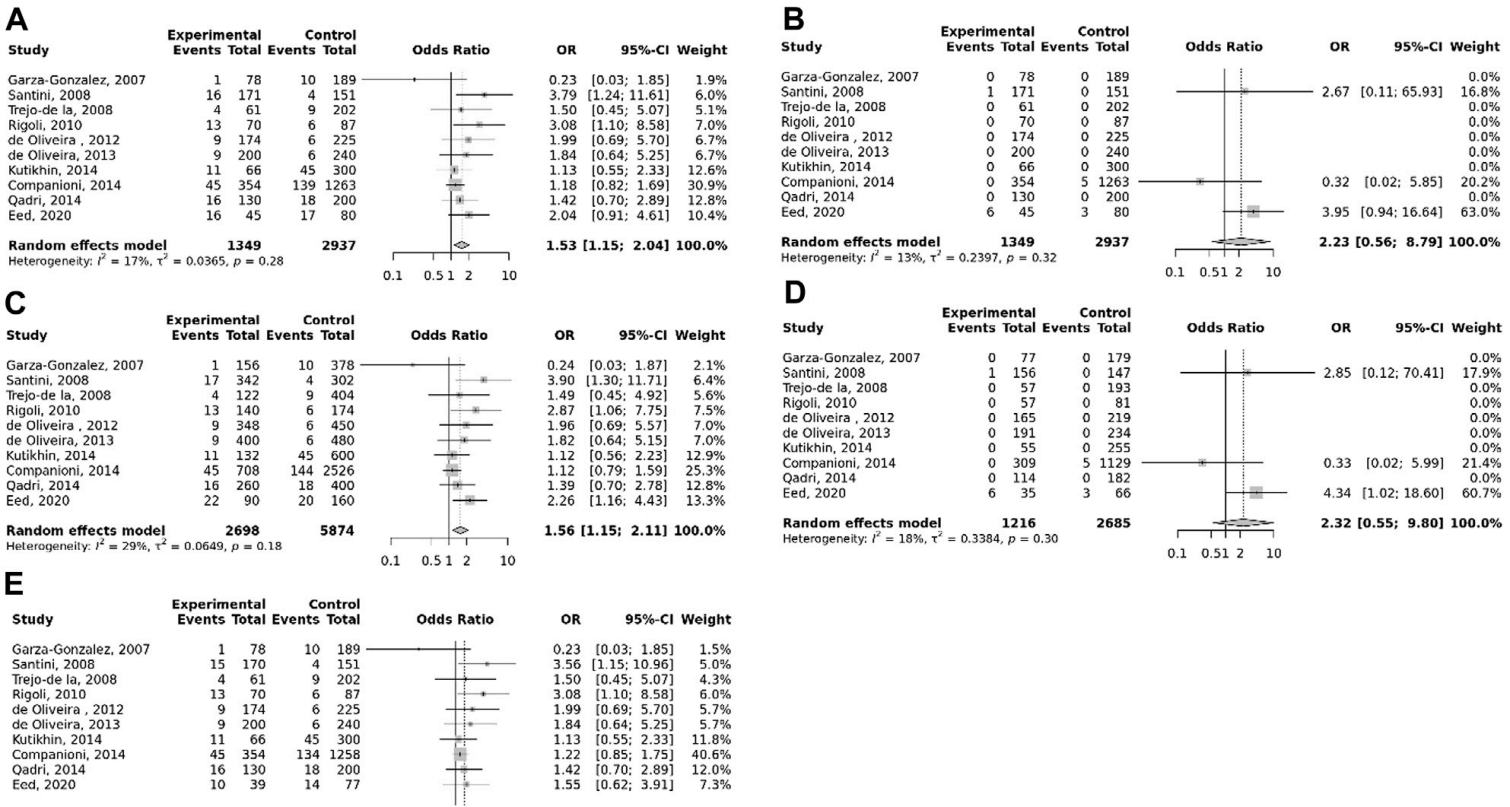
Meta-analysis of the association between TLR-4 rs4986791 C<T polymorphism with gastric cancer in dominant **(A)**, recessive **(B)**, allelic **(C)**, homozygote (TT vs. CC) **(D)**, and heterozygote (CT vs. CC) **(E)** models.

**FIGURE 4:**
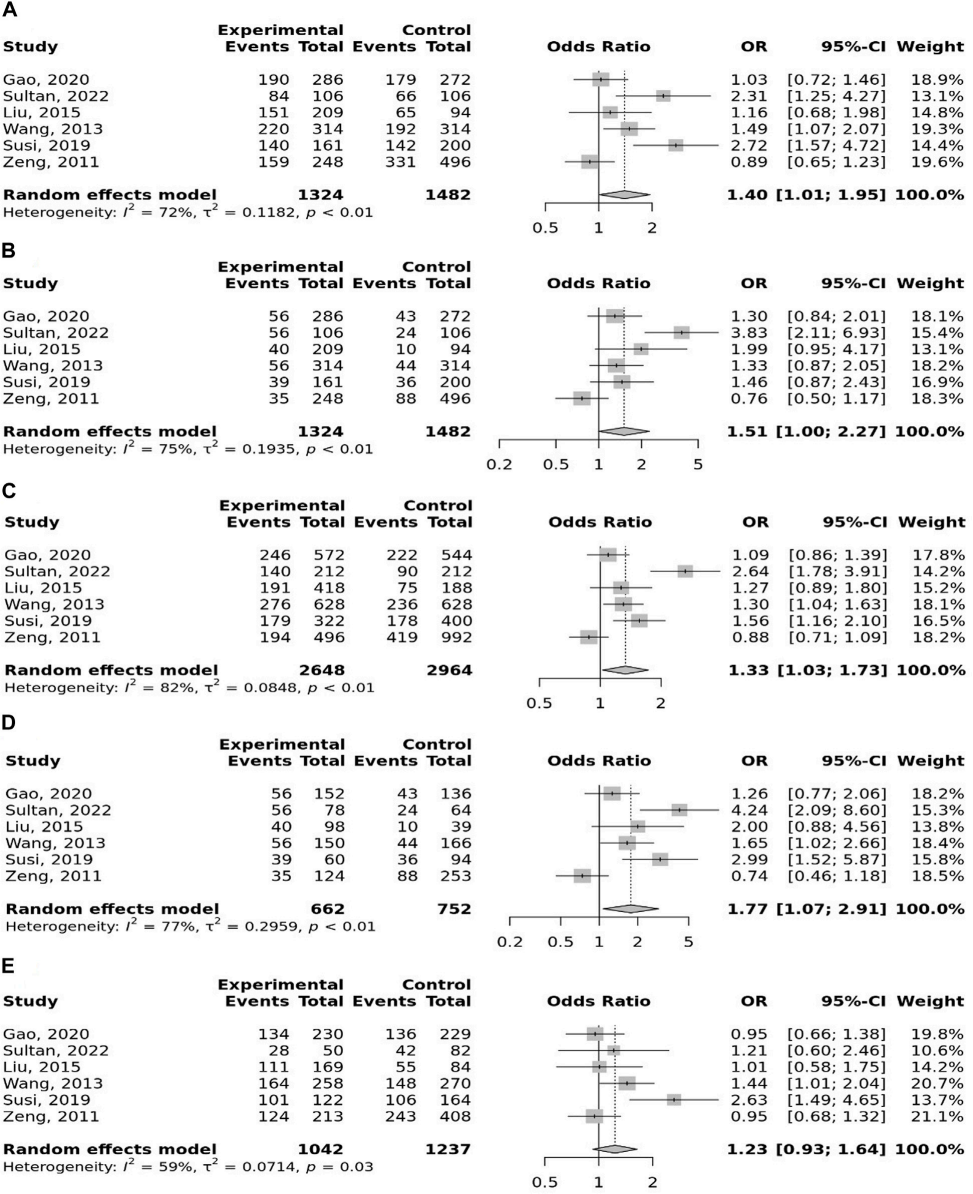
Meta-analysis of the association between TLR-9 rs187084 T>C polymorphism with gastric cancer in dominant **(A)**, recessive **(B)**, allelic **(C)**, homozygote (CC vs. TT) **(D)**, and heterozygote (TC vs. TT) **(E)** models.

The overall pooled analyses and subgroup analyses by ethnicity are reported in Table 2. In the overall analyses, TLR-4 rs4986790 was significantly associated with an increased risk of GC (dominant model: OR = 1.51, 95% CI: 1.12–2.03; recessive model: OR = 2.58, 95% CI: 1.06–6.30; allelic model: OR = 1.50, 95% CI: 1.12–2.01; homozygote model: OR = 2.58, 95% CI: 1.05–6.34; and heterozygote model (AG vs. AA): OR = 1.46, 95% CI: 1.09–1.97) (Figure 2). An increased odds of GC was also found for variations in TLR-4 rs4986791 (dominant model: OR = 1.53, 95% CI: 1.15–2.04; allelic model: OR = 1.56, 95% CI: 1.15–2.11; heterozygote model (CT vs. CC): OR = 1.45, 95% CI: 1.12–1.87) (Figure 3). Additionally, the analysis revealed a significant association between TLR-9 rs187084 and GC risk (dominant model: OR = 1.40, 95% CI: 1.01–1.95; recessive model: OR = 1.51, 95% CI: 1.003–2.27; allelic model: OR = 1.33, 95% CI: 1.03–1.73; homozygote model (CC vs. TT): OR = 1.77, 95% CI: 1.07–2.91) (Figure 4). No significant association with GC risk was observed for TLR-2 -196 to -174de (Delta22), TLR-2 rs3804100, TLR-4 rs11536889, TLR-4 rs11536878, TLR-4 rs2770150, TLR-4 rs10116253, TLR-4 rs1927911, TLR-4 rs10983755, TLR-4 rs10759932, TLR-4 rs1927914, TLR-9 rs352140, and TLR-10 rs10004195 (Table 2).

**TABLE 2:** Main results and subgroup analysis by ethnicity for the meta-analysis of various types of toll-like receptors and gastric cancer susceptibility.

				Test of association		Test of heterogeneity		Egger’s test of publication bias
Type of toll-like receptors	Genetic model	Subgroups	Effect sizes	OR	95% CI	I^2^ (%)	*p*	*p*
TLR-1 rs4833095 T>C	Dominant model	Overall	3	0.54	0.08–3.69	98.0	0.01	0.61
	Recessive model	Overall	3	0.66	0.31–1.39	55.0	0.11	0.21
	Allelic model	Overall	3	0.64	0.18–2.34	97.0	0.01	0.54
	CC vs. TT	Overall	3	0.53	0.10–2.92	91.0	0.01	0.03
	CT vs. TT	Overall	3	0.50	0.07–3.50	97.0	0.01	0.53
TLR-2 -196 to - 174de (Delta22)	Dominant model	Overall	11	1.31	0.91–1.89	89.0	0.01	0.67
		Asian	7	1.19	0.92–1.55	68.0	0.004	
		Caucasian	1	1.25	0.64–2.45	-	-	
		Latino	3	1.60	0.39–6.44	96.0	0.01	
		60 years ≤	6	1.40	0.67–2.90	92.0	0.01	
		60 years >	5	1.19	0.85–1.66	78.0	0.04	
		Community-based	6	1.30	0.81–2.09	86.0	0.01	
		Hospital-based	5	1.32	0.69–2.51	92.0	0.01	
	Recessive model	Overall	11	1.34	0.85–2.10	75.0	0.01	0.34
		Asian	7	1.17	0.73–1.86	77.0	0.01	
		Caucasian	1	3.02	0.34–26.48	-	-	
		Latino	3	1.97	0.34–11.28	82.0	0.003	
		60 years ≤	6	1.87	0.74–4.76	79.0	0.04	
		60 years >	5	1.06	0.64–1.75	72.0	0.006	
		Community-based	6	1.52	0.75–3.10	73.0	0.001	
		Hospital-based	5	1.08	0.62–1.88	71.0	0.007	
	Allelic model	Overall	11	1.25	0.93–1.69	90.0	0.01	0.53
		Asian	7	1.14	0.91–1.42	78.0	0.01	
		Caucasian	1	1.32	0.73–2.39	-	-	
		Latino	3	1.53	0.43–5.39	96.0	0.01	
		60 years ≤	6	1.35	0.74–2.49	93.0	0.01	
		60 years >	5	1.13	0.87–1.47	81.0	0.04	
		Community-based	6	1.27	0.85–1.90	87.0	0.01	
		Hospital-based	5	1.22	0.74–2.01	93.0	0.01	
	Del/del vs. ins/ins	Overall	11	1.51	0.89–2.56	78.0	0.01	0.42
		Asian	7	1.29	0.75–2.21	0.77	0.01	
		Caucasian	1	3.14	0.35–27.69	-	-	
		Latino	3	2.24	0.28–17.91	87.0	0.01	
		60 years ≤	6	2.14	0.79–5.78	76.0	0.04	
		60 years >	5	1.13	0.62–2.06	78.0	0.04	
		Community-based	6	1.68	0.80–3.52	74.0	0.001	
		Hospital-based	5	1.31	0.60–2.86	79.0	0.001	
	Ins/del vs. ins/ins	Overall	11	1.27	0.89–1.80	86.0	0.01	0.71
		Asian	7	1.15	0.90–1.46	58.0	0.02	
		Caucasian	1	1.14	0.57–2.28	-	-	
		Latino	3	1.54	0.41–5.82	96.0	0.01	
		60 years ≤	6	1.32	0.65–2.70	91.0	0.01	
		60 years >	5	1.18	0.87–1.60	71.0	0.006	
		Community-based	6	1.21	0.76–1.92	84.0	0.01	
		Hospital-based	5	1.33	0.72–2.46	90.0	0.01	
TLR-2 rs3804099 T>C	Dominant model	Overall	4	0.82	0.61–1.09	31.0	0.46	0.13
		Asian	1	0.68	0.49–0.92	-	-	
		Caucasian	1	0.87	0.43–1.72	-	-	
		Latino	2	0.93	0.50–1.73	76.0	0.03	
		Community-based	3	0.89	0.57–1.38	60.0	0.08	
		Hospital-based	1	0.69	0.52–1.12	-	-	
	Recessive model	Overall	4	0.83	0.65–1.06	0.0	0.59	0.17
		Asian	1	0.67	0.37–1.19	-	-	
		Caucasian	1	0.88	0.40–1.91	-	-	
		Latino	2	0.87	0.66–1.16	18.0	0.26	
		Community-based	3	0.88	0.67–1.14	0.0	0.56	
		Hospital-based	1	0.67	0.44–1.04	-	-	
	Allelic model	Overall	4	0.84	0.70–1.01	42.0	0.15	0.81
		Asian	1	0.73	0.58–0.93	-	-	
		Caucasian	1	0.91	0.59–1.39	-	-	
		Latino	2	0.88	0.64–1.22	70.0	0.06	
		Community-based	3	0.88	0.75–1.03	50.0	0.13	
		Hospital-based	1	0.73	0.59–1.11	-	-	
	CC vs. TT	Overall	4	0.76	0.50–1.16	42.0	0.15	0.65
		Asian	1	0.56	0.31–1.02	-	-	
		Caucasian	1	0.81	0.32–2.03	-	-	
		Latino	2	0.85	0.39–1.83	73.0	0.05	
		Community-based	3	0.87	0.60–1.25	47.0	0.14	
		Hospital-based	1	0.60	0.39–1.04	-	-	
	CT vs. TT	Overall	4	0.84	0.63–1.13	42.0	0.15	0.42
		Asian	1	0.70	0.50–0.96	-	-	
		Caucasian	1	0.89	0.43–1.82	-	-	
		Latino	2	0.98	0.52–1.83	74.0	0.04	
		Community-based	3	0.92	0.59–1.45	58.0	0.08	
		Hospital-based	1	0.73	0.53–1.009	-	-	
TLR-2 rs3804100 T>C	Dominant model	Overall	3	1.11	0.79–1.55	0.0	0.51	0.33
		Asian	2	1.01	0.70–1.47	0.0	0.67	
		Caucasian	1	1.65	0.74–3.65	-	-	
	Recessive model	Overall	3	0.82	0.22–3.10	0.40	0.19	0.84
		Asian	2	0.55	0.19–1.60	9.0	0.29	
		Caucasian	1	2.78	0.44–17.33	-	-	
	Allelic model	Overall	3	1.04	0.73–1.50	32.0	0.23	0.51
		Asian	2	0.92	0.67–1.26	0.0	0.49	
		Caucasian	1	1.67	0.85–3.30	-	-	
	CC vs. TT	Overall	3	0.87	0.22–3.47	43.0	0.17	0.84
		Asian	2	0.58	0.19–1.70	12.0	0.28	
		Caucasian	1	3.10	0.49–19.60	-	-	
	CT vs. TT	Overall	3	1.18	0.84–1.68	0.0	0.83	
		Asian	2	1.12	0.77–1.65	0.0	0.88	
		Caucasian	1	1.49	0.64–3.46	-	-	
TLR-4 rs11536889 G>C	Dominant model	Overall	8	1.01	0.90–1.12	0.0	0.67	0.14
		Asian	5	1.23	0.83–1.83	29.6	0.22	
		Caucasian	3	1	0.73–1.35	0.0	0.92	
		60 years ≤	4	1.03	0.83–1.27	21.0	0.28	
		60 years >	4	1.00	0.87–1.14	0.0	0.67	
		Community-based	1	1.08	0.64–1.83	-	-	
		Hospital-based	7	1.00	0.89–1.12	0.0	0.50	
	Recessive model	Overall	8	1.08	0.85–1.38	0.0	0.48	0.49
		Asian	5	1.23	0.83–1.83	29.6	0.22	
		Caucasian	3	1.00	0.73–1.35	0.0	0.92	
		60 years ≤	4	1.32	0.71–2.47	0.48	0.12	
		60 years >	4	1.01	0.75–1.36	0.0	0.97	
		Community-based	1	1.31	0.23–7.31	-	-	
		Hospital-based	7	1.08	0.83–1.42	7.0	0.32	
	Allelic model	Overall	8	1.02	0.93–1.12	7.0	0.37	0.19
		Asian	5	1.03	0.90–1.17	40.0	0.15	
		Caucasian	3	1.01	0.89–1.13	0.0	0.67	
		60 years ≤	4	1.08	0.85–1.3	53.0	0.09	
		60 years >	4	1.00	0.89–1.12	0.0	0.80	
		Community-based	1	1.08	0.69–1.69	-	-	
		Hospital-based	7	1.02	0.91–1.14	19.0	0.27	
	CC vs. GG	Overall	8	1.07	0.83–1.37	6.9	0.37	0.42
		Asian	5	1.23	0.83–1.84	40.1	0.15	
		Caucasian	3	0.98	0.71–1.34	0.0	0.97	
		60 years ≤	4	1.35	0.68–2.68	55.0	0.07	
		60 years >	4	0.99	0.73–1.35	0.0	0.98	
		Community-based	1	1.34	0.23–7.57	-	-	
		Hospital-based	7	1.09	0.80–1.49	19.0	0.28	
	GC vs. GG	Overall	8	1.00	0.89–1.12	0.0	0.81	0.17
		Asian	5	0.99	0.84–1.16	0.0	0.76	
		Caucasian	3	1.01	0.86–1.17	0.0	0.40	
		60 years ≤	4	0.99	0.82–1.19	0.0	0.61	
		60 years >	4	1.00	0.86–1.15	0.0	0.59	
		Community-based	1	1.07	0.62–1.82	-	-	
		Hospital-based	7	0.99	0.88–1.11	0.0	0.72	
TLR-4 rs4986790 (+896A <G)	Dominant model	Overall	14	1.51	1.12–2.03	55.0	0.01	0.64
		Asian	2	0.57	0.09–3.62	69.0	0.07	
		Caucasian	8	1.48	1.003–2.19	64.0	0.01	
		Latino	4	2.01	1.35–3.15	0.0	0.47	
		60 years ≤	4	1.93	1.21–3.09	20.0	0.29	
		60 years >	5	1.22	0.92–1.61	0.0	0.42	
		Community-based	8	1.71	1.11–2.63	63.0	0.01	
		Hospital-based	6	1.23	0.89–1.70	16.0	0.31	
	Recessive model	Overall	7	2.58	1.06–6.30	0.0	0.54	0.58
		Asian	1	0.53	0.03–11.21	-	-	
		Caucasian	6	2.99	1.18–7.60	0.0	0.57	
		60 years ≤	1	2.67	0.11–65.93	-	-	
		60 years >	5	2.21	0.58–8.41	21.0	0.28	
		Community-based	4	1.98	0.48–8.15	0.0	0.81	
		Hospital-based	3	1.82	0.37–12.08	48.0	0.15	
	Allelic model	Overall	14	1.50	1.12–2.01	57.0	0.01	0.62
		Asian	2	0.53	0.08–3.68	72.0	0.06	
		Caucasian	8	1.49	1.02–2.18	66.0	0.01	
		Latino	4	1.99	1.32–3.02	0.0	0.48	
		60 years ≤	4	1.91	1.27–2.87	3.0	0.38	
		60 years >	5	1.26	0.84–1.88	42.0	0.14	
		Community-based	8	1.66	1.10–2.48	62.0	0.01	
		Hospital-based	6	1.30	0.86–1.95	46.0	0.10	
	GG vs. AA.	Overall	7	2.58	1.05–6.34	0.0	0.53	0.97
		Asian	1	0.50	0.02–10.48	-	-	
		Caucasian	6	3.02	1.18–7.73	0.0	0.57	
		60 years ≤	1	2.64	0.11–65.39	-	-	
		60 years >	5	2.22	0.59–8.40	20.0	0.29	
		Community-based	4	2.05	0.50–8.44	0.0	0.78	
		Hospital-based	3	1.78	0.26–12.09	48.0	0.14	
	AG vs. AA.	Overall	14	1.46	1.09–1.97	54.0	0.01	0.58
		Asian	2	0.63	0.12–3.39	64.0	0.10	
		Caucasian	8	1.40	0.94–2.09	63.0	0.01	
		Latino	4	2.06	1.35–3.15	0.0	0.47	
		60 years ≤	4	1.90	1.14–3.16	30.0	0.23	
		60 years >	5	1.17	0.88–1.56	0.0	0.53	
		Community-based	8	1.71	1.11–2.63	62.0	0.01	
		Hospital-based	6	1.18	0.90–1.54	0.0	0.5	
TLR-4 rs4986791 C < T	Dominant model	Overall	10	1.53	1.15–2.04	17.0	0.28	0.36
		Asian	1	1.42	0.70–2.89	-	-	
		Caucasian	5	1.69	1.08–2.64	45.0	0.12	
		Latino	4	1.46	0.75–2.86	15.0	0.32	
		60 years ≤	4	2.15	1.24–3.73	0.0	0.70	
		60 years >	4	1.22	0.81–1.85	27.0	0.25	
		Community-based	5	1.93	1.24–3.02	7.0	0.37	
		Hospital-based	5	1.29	0.94–1.76	6.0	0.38	
	Recessive model	Overall	3	2.23	0.56–8.79	13.0	0.32	0.19
	Allelic model	Overall	10	1.56	1.15–2.11	29.0	0.18	0.35
		Asian	1	1.39	0.70–2.78	-	-	
		Caucasian	5	1.73	1.08–2.77	57.0	0.06	
		Latino	4	1.46	0.76–2.80	13.0	0.33	
		60 years ≤	4	2.15	1.25–3.70	0.0	0.66	
		60 years >	4	1.25	0.77–2.03	50.0	0.11	
		Community-based	5	1.91	1.22–2.99	12.0	0.34	
		Hospital-based	5	1.34	0.90–2.00	33.0	0.0	
	TT vs. CC	Overall	3	2.32	0.55–9.80	18.0	0.30	0.17
	CT vs. CC	Overall	10	1.45	1.12–1.87	4.0	0.40	0.45
		Asian	1	1.42	0.70–2.89	-	-	
		Caucasian	5	1.56	1.05–2.33	31.0	0.21	
		Latino	4	1.46	0.75–2.86	15.0	0.32	
		60 years ≤	4	2.11	1.22–3.67	0.0	0.75	
		60 years >	4	1.19	0.88–1.61	0.0	0.43	
		Community-based	5	1.89	1.23–2.91	1.0	0.40	
		Hospital-based	5	1.26	0.94–1.68	0.0	0.56	
TLR-4 rs11536878 C>A	Dominant model	Overall	3	1.01	0.85–1.19	0.0	0.94	0.76
	Recessive model	Overall	2	0.97	0.39–2.36	0.0	0.95	-
	Allelic model	Overall	2	0.99	0.78–1.25	0.0	0.80	-
	AA vs. CC	Overall	2	0.97	0.39–2.73	0.0	0.96	-
	CA vs. CC	Overall	2	0.99	0.76–1.29	0.0	0.74	-
TLR-4 rs2770150 T>C	Dominant model	Overall	2	0.82	0.45–1.51	0.0	0.57	-
	Recessive model	Overall	2	1.29	0.94–1.78	-	-	-
	Allelic model	Overall	2	1.13	0.88–1.46	0.0	0.44	-
	TT vs. CC	Overall	2	0.98	0.52–1.85	-	-	-
	CT vs. CC	Overall	2	0.70	0.37–1.32	0.0	0.64	-
TLR-4 rs10116253 T>C	Dominant model	Overall	3	0.77	0.57–1.04	0.43	0.17	0.39
	Recessive model	Overall	3	0.81	0.43–1.52	77.0	0.01	0.91
	Allelic model	Overall	3	0.52	0.60–1.11	72.0	0.03	0.71
	CC vs. TT	Overall	3	0.71	0.36–1.39	76.0	0.02	0.78
	CT vs. TT	Overall	3	0.81	0.64–1.03	5.0	0.35	0.05
TLR-4 rs1927911 C>T	Dominant model	Overall	3	0.74	0.49–1.12	73.0	0.03	0.05
	Recessive model	Overall	3	0.71	0.41–1.22	71.0	0.03	0.66
	Allelic model	Overall	3	0.77	0.56–1.07	78.0	0.02	0.31
	TT vs. CC	Overall	3	0.63	0.33–1.19	74.0	0.02	0.47
	CT vs. CC	Overall	3	0.80	0.53–1.19	66.0	0.05	0.17
TLR-4 rs10983755 G>A	Dominant model	Overall	3	0.93	0.73–1.19	68.0	0.04	0.64
	Recessive model	Overall	2	1.12	0.80–1.57	0.0	0.44	-
	Allelic model	Overall	2	0.95	0.70–1.29	76.0	0.04	-
	AA vs. GG	Overall	2	1.04	0.65–1.64	43.0	0.18	-
	GA vs. GG	Overall	2	0.85	0.54–1.32	80.0	0.03	-
TLR-4 rs10759932 T>C	Dominant model	Overall	6	0.77	0.59–1.003	75.0	0.07	0.18
	Recessive model	Overall	6	1.28	0.73–2.27	82.0	0.37	0.80
	Allelic model	Overall	6	0.88	0.72–1.08	72.0	0.01	0.34
	CC vs. TT	Overall	6	1.15	0.66–2.02	80.0	0.01	0.17
	TC vs. TT	Overall	6	0.80	0.61–1.05	70.0	0.01	0.15
TLR-4 rs1927914 T>C	Dominant model	Overall	2	0.92	0.73–1.17	54.0	0.14	-
	Recessive model	Overall	2	0.94	0.67–1.31	59.0	0.12	-
	Allelic model	Overall	2	0.94	0.77–1.15	70.0	0.07	-
	CC vs. TT	Overall	2	0.89	0.57–1.37	71.0	0.06	-
	TC vs. TT	Overall	2	0.94	0.79–1.11	6.0	0.30	
TLR-5 rs5744174 T>C	Dominant model	Overall	3	1.21	0.99–1.49	30.0	0.24	0.56
		Asian	2	1.26	1.09–1.45	0.0	0.59	
		Caucasian	1	0.74	0.39–1.39	-	-	
	Recessive model	Overall	3	1.71	1.26–2.31	0.0	0.77	0.06
		Asian	2	1.65	1.20–2.28	0.0	0.64	
		Caucasian	1	2.16	0.87–5.36	-	-	
	Allelic model	Overall	3	1.25	1.11–1.40	0.0	0.65	0.79
		Asian	2	1.26	1.12–1.42	0.0	0.58	
		Caucasian	1	1.06	0.68–1.64	-	-	
	CC vs. TT	Overall	3	1.74	1.28–2.37	0.0	0.85	0.74
		Asian	2	1.75	1.27–2.43	0.0	0.58	
		Caucasian	1	1.60	0.60–4.29	-	-	
	TC vs. TT	Overall	3	1.09	0.82–1.46	56.0	0.10	0.49
		Asian	2	1.19	1.03–1.39	0.0	0.64	
		Caucasian	1	0.58	0.29–1.12	-	-	
TLR-9 -1237 T>C (rs5743836)	Dominant model	Overall	2	1.22	0.56–2.64	72.0	0.06	0.81
	Recessive model	Overall	2	2.05	0.70–6.02	0.0	0.48	-
	Allelic model	Overall	2	1.30	0.61–2.75	75.0	0.05	-
	CC vs. TT	Overall	2	2.02	0.69–5.93	0.0	0.42	-
	TC vs. TT	Overall	2	1.10	0.55–2.23	66.0	0.09	-
TLR-9 −2848 G>A (rs352140)	Dominant model	Overall	2	0.95	0.68–1.33	0.0	0.88	-
	Recessive model	Overall	2	0.96	0.61–1.52	0.0	0.51	-
	Allelic model	Overall	2	0.96	0.74–1.23	0.0	0.71	-
	AA vs. GG	Overall	2	0.94	0.59–1.52	0.0	0.52	-
	AG vs. GG	Overall	2	0.95	0.65–1.39	0.0	0.95	-
TLR-9 rs187084 T>C	Dominant model	Overall	6	1.40	1.01–1.95	72.0	0.01	0.11
		Asian	4	1.11	0.87–1.41	40.0	0.16	
		Caucasian	1	2.31	1.25–4.26	-	-	
		Latino	1	2.72	1.56–4.72	-	-	
		60 years ≤	2	1.93	1.07–3.47	70.0	0.06	
		60 years >	4	1.16	0.82–1.64	60.0	0.05	
		Community-based	3	1.23	0.99–1.53	13.0	0.31	
		Hospital-based	3	1.72	0.78–3.79	87.0	0.04	
	Recessive model	Overall	6	1.51	1.003–2.27	75.0	0.01	0.12
		Asian	4	1.20	0.84–1.71	53.0	0.09	
		Caucasian	1	3.82	2.11–6.92	-	-	
		Latino	1	1.45	0.87–2.42	-	-	
		60 years ≤	2	1.38	0.99–1.92	0.0	0.79	
		60 years >	4	1.60	0.81–3.17	84.0	0.04	
		Community-based	3	1.39	1.05–1.85	0.0	0.59	
		Hospital-based	3	1.58	0.64–3.89	89.0	0.04	
	Allelic model	Overall	6	1.33	1.03–1.73	82.0	0.01	0.06
		Asian	4	1.10	0.91–1.33	55.0	0.07	
		Caucasian	1	2.63	1.77–3.90	-	-	
		Latino	1	1.56	1.16–2.09	-	-	
		60 years ≤	2	1.39	1.16–1.66	0.0	0.33	
		60 years >	4	1.30	0.87–1.94	87.0	0.01	
		Community-based	3	1.21	1.04–1.40	0.0	0.56	
		Hospital-based	3	1.50	0.81–2.78	92.0	0.01	
	CC vs. TT	Overall	6	1.77	1.07–2.91	77.0	0.01	0.09
		Asian	4	1.26	0.82–1.92	59.0	0.06	
		Caucasian	1	4.24	2.09–8.59	-	-	
		Latino	1	2.99	1.52–5.87	-	-	
		60 years ≤	2	2.11	1.19–3.75	49.0	0.15	
		60 years >	4	1.60	0.78–3.30	82.0	0.04	
		Community-based	3	1.51	1.10–2.08	0.0	0.57	
		Hospital-based	3	2.05	0.65–6.47	90.0	0.01	
	TC vs. TT	Overall	6	1.23	0.93–1.64	59.0	0.03	0.44
		Asian	4	1.08	0.87–1.33	18.0	0.29	
		Caucasian	1	1.21	0.59–2.45	-	-	
		Latino	1	2.63	1.49–4.64	-	-	
		60 years ≤	2	1.86	1.03–3.34	68.0	0.07	
		60 years >	4	0.98	0.79–1.21	0.0	0.93	
		Community-based	3	1.15	0.91–1.45	27.0	0.25	
		Hospital-based	3	1.41	0.73–2.71	78.0	0.01	
TLR-10 rs10004195 A <T	Dominant model	Overall	2	1.02	0.55–1.9	0.0	0.87	0.90
	Recessive model	Overall	2	1.42	0.71–2.81	0.0	0.48	0.64
	Allelic model	Overall	2	1.16	0.75–1.79	0.0	0.58	0.65
	TT vs. AA	Overall	2	1.31	0.61–2.82	0.0	0.58	0.92
	AT vs. AA	Overall	2	0.84	0.41–1.72	0.0	0.084	0.77

### Subgroup analyses

**FIGURE 5:**
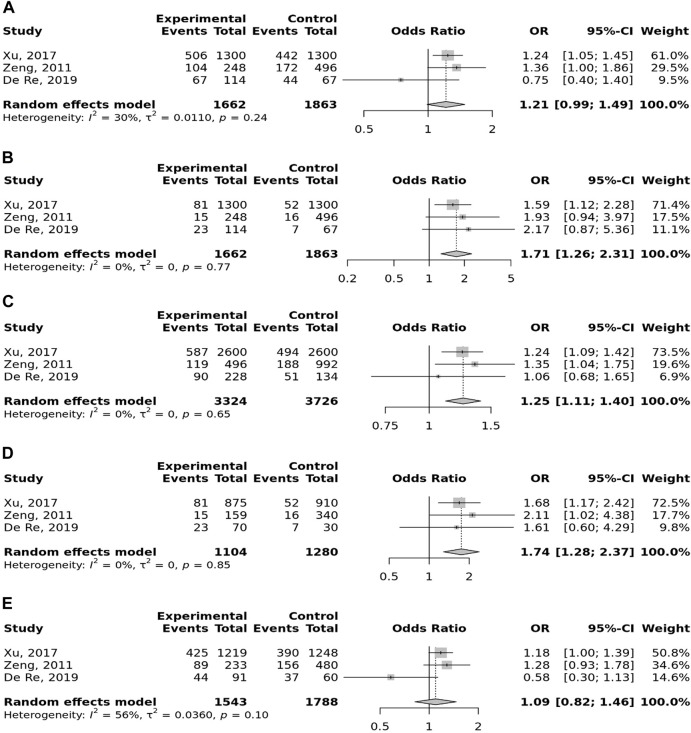
Meta-analysis of the association between TLR-5 rs5744174 T>C polymorphism with gastric cancer in dominant **(A)**, recessive **(B)**, allelic **(C)**, homozygote (CC vs. TT) **(D)**, and heterozygote (TC vs. TT) **(E)** models.

In the stratified analysis, the association of TLR-4 rs4986790 with GC was supported by the subgroups of Caucasian, Latino, people of age ≤60 years, and studies with the community-based control group (Table 2). Moreover, the association of TLR-4 rs4986791 with GC was supported by the subgroups of Caucasian, people of age ≤60 years, and studies with the community-based control group (Table 2). In different genetic models, there was a positive relationship between TLR-5 rs5744174 and GC susceptibility (recessive model: OR = 1.71, 95% CI: 1.26–2.31; allelic model: OR = 1.25, 95% CI: 1.11–1.40; homozygote model (CC vs. TT): OR = 1.74, 95% CI: 1.28–2.37) (Figure 5), which was also observed in the Asian population (Table 2). In the stratified analysis, the association of TLR-9 rs187084 with GC was supported by the subgroups of Caucasian, Latino, people of age ≤60 years, and studies with the community-based control group (Table 2).

### Heterogeneity and publication bias

For the SNPs of TLR-2 rs3804099, TLR-2 rs3804100, TLR-4 rs11536889, TLR-4 rs4986791, TLR-4 rs11536878, TLR-4 rs2770150, TLR-5 rs5744174, and TLR-9 rs352140, no significant heterogeneity was detected across the studies, but the test of heterogeneity was significant for other investigated SNPs (Table 2). Egger’s test of publication bias revealed no evidence of publication bias for most SNPs (Table 2). Funnel plots for publication bias for SNPs with ≥10 studies under the dominant model are given in Supplementary File S2.

## Discussion

This meta-analysis examined the association of 18 common SNPs in the TLR family to GC risk. The results indicated that variations in TLR-4 rs4986790, TLR-4 rs4986791, TLR-5 rs5744174, and TLR-9 rs187084 were significantly associated with increased risk of GC in different genetic models.

Given the close relationship between inflammation and carcinogenesis, several studies have attempted to disclose the role of TLRs in GC progression. However, the available data were contradictory. The protein function of TLRs might be damaged by SNPs in genes coding TLRs, resulting in altered vulnerability to malignancies (Wang et al., 2023). Previous meta-analyses on the relation of TLR SNPs to GC have only focused on four common SNPs in the TLR family, namely, TLR-2-196 to -174de (Delta22), TLR-4 rs11536889, TLR-4 rs4986790, and TLR-4 rs498679, with inconclusive results. While the study by Castano-Rodriguez indicated a significant association of TLR-2-196 to -174de (Delta22) with GC (Castaño-Rodríguez et al., 2013), the meta-analysis by Chen et al. (2013) failed to find an association. In line with our findings, a previous meta-analysis by Zhao et al. identified a significant direct association between TLR-4 rs4986790 and GC, while, in contrast to the present meta-analysis, no association was found for TLR-4 rs4986791 (Zhao et al., 2013). Confirming our results, the significant relation of TLR-4 rs4986790 and TLR-4 rs4986791 variations to GC has been proposed by another meta-analysis (Zhou et al., 2014). Moreover, similar to our study, two previous meta-analyses by Wang X. et al. (2015) and Chen et al. (2013) did not find a significant relationship between TLR-4 rs11536889 and GC. The present study is the first meta-analysis showing a significant relationship between TLR-5 rs5744174 and TLR-9 rs187084 and genetic susceptibility to GC. The differences in the results of the previous meta-analyses may be due to the small number of the analyzed studies and, thus lack of sufficient statistical power to detect the true associations.

Mechanistically, genetic variations in TLRs may result in impaired TLR signaling and alter their affinity to the ligands, leading to variations in immune responses of the gastric mucosa against carcinogens and dysregulated inflammation, leading to modulation of *H. pylori* infection and, thus, GC risk (Zhou et al., 2014; Tongtawee et al., 2019). TLR signaling plays a crucial role in shaping the tumor microenvironment (Wang et al., 2023). Polymorphisms in TLR genes could influence the recruitment and activation of immune cells, tumor-associated inflammation, and immune evasion mechanisms employed by cancer cells, which all affect cancer susceptibility (Paulos et al., 2007). TLR-2 activation results in the activation of nuclear factor-κB (NF-κB) and functions as an innate immune response (de Matos Lourenço et al., 2020). It has been suggested that NF-κB is a main mediator of inflammation-induced tumor progression (Wang et al., 2023). The rs3804099 variants may reduce TLR-2-related chronic inflammation and induce apoptosis (Kim and Karin, 2011; Semlali et al., 2017; Mohamed et al., 2020), resulting in decreased risk of GC. Furthermore, the TLR-5 rs5744174 may affect the recognition and binding affinity of TLR-5 toward bacterial flagellin, leading to compromised antibacterial responses and prolonged exposure to pathogens, thus promoting gastric oncogenesis (Castaño-Rodríguez et al., 2014). TLR-9 impacts tumor development and progression through various molecular mechanisms, such as activation of immune response, induction of apoptosis, modulation of angiogenesis, and regulation of tumor cell proliferation and survival. TLR-9 activation in GC cells leads to the production of pro-inflammatory cytokines and chemokines, such as TNF-alpha, IL-6, and IL-8 (Karapetyan et al., 2020). These molecules attract immune cells, such as macrophages and dendritic cells, to the tumor microenvironment. This immune response helps in tumor recognition, elimination, and control (Karapetyan et al., 2020). TLR-9 activation can induce apoptosis in GC cells. This is mediated by the activation of caspases, which are enzymes involved in the execution of apoptosis (Sindhava et al., 2017). TLR-9 can influence the angiogenesis that is required for tumor growth and metastasis. Studies have shown that TLR-9 activation can inhibit angiogenesis by suppressing the production of pro-angiogenic factors, such as vascular endothelial growth factor (VEGF) (He et al., 2018b). Activation of TLR-9 impacts the proliferation and survival of GC cells (Tang et al., 2022). It can activate signaling pathways, such as the nuclear factor kappa-light-chain-enhancer of activated B-cell (NF-κB) pathway, which is involved in promoting cell proliferation and survival (Tsujimura et al., 2004). Regarding TLR-5, induction of TLR-5 leads to the production of pro-inflammatory cytokines and chemokines, such as interleukin 1 beta (IL-1β), IL-6, and IL-8. These molecules attract immune cells, including macrophages and natural killer cells, to the tumor microenvironment. This immune response aids in tumor recognition and elimination (Tallant et al., 2004). TLR-5 plays a role in the integrity and barrier function of the epithelial layer in the stomach (Feng et al., 2023). This is achieved through the activation of various signaling pathways, such as the NF-κB pathway (Tallant et al., 2004), which regulates the expression of genes involved in tight junction formation and epithelial cell adhesion (Al-Sadi et al., 2016). A well-functioning epithelial barrier helps prevent the infiltration of potentially harmful agents and pathogens into the underlying tissues (Mitamura et al., 2021). TLR-5 activation has been shown to affect the invasive potential and metastatic spread of GC cells (Castaño-Rodríguez et al., 2014). This effect is mediated by the suppression of various molecular pathways involved in invasive behavior, such as the matrix metalloproteinase (MMP) family enzymes that degrade the extracellular matrix (Jiang et al., 2017), a critical step in tumor invasion and metastasis (Zhuyan et al., 2020). Studies have also demonstrated that activation of TLR-5 promotes apoptosis in cancer cells through the activation of caspases and the upregulation of pro-apoptotic factors (Zeng et al., 2006). This helps in reducing tumor burden and inhibiting tumor progression. Upon binding to molecular patterns, TLR-4 triggers the activation of inflammatory pathways, leading to the release of pro-inflammatory cytokines, chemokines, and various immune cells (Escoubet-Lozach et al., 2011). Chronic inflammation can promote tumor growth, angiogenesis, and metastasis in GC (Landskron et al., 2014). TLR-4 signaling can activate the NF-κB pathway (Verstrepen et al., 2008). Activation of NF-κB promotes the expression of genes involved in cell survival, proliferation, and angiogenesis, which can contribute to the development and progression of GC (Rastogi et al., 2023). TLR-4 activation has been shown to induce epithelial–mesenchymal transition (EMT), a process in which epithelial cells acquire a mesenchymal phenotype (Shi et al., 2016). EMT is associated with increased invasive potential and metastasis in cancer cells (Huang et al., 2015). TLR-4-induced EMT in GC is mediated through signaling pathways such as Snail, Twist, and ZEB1, which are known regulators of EMT markers (Jing et al., 2012). TLR-4 activation can contribute to the activation of cancer-associated fibroblasts (CAFs) and the development of fibrosis in the GC microenvironment (Janovec et al., 2021). CAFs can promote GC progression through the secretion of growth factors, extracellular matrix remodeling, and immune modulation (Kobayashi et al., 2019). TLR-4 signaling can directly impact tumor cell behavior. Activation of TLR-4 promotes cell survival and proliferation through signaling pathways such as phosphatidylinositol 3-kinase (PI3K)/protein kinase B (AKT), mitogen-activated protein kinase/extracellular signal-regulated kinase (MAPK/ERK), and the Janus kinase (JAK)/signal transducer and activator of transcription (STAT) (JAK/STAT) (Vivarelli et al., 2004; Korneev et al., 2017). These pathways regulate various aspects of cell growth, survival, and apoptosis resistance, ultimately influencing GC cell behavior (Hu et al., 2014; Sun et al., 2015). It is important to note that the molecular mechanisms of TLRs in GC are still an area of active research, and further studies are needed to fully understand the complexities of the TLR-mediated effects on GC development and progression. Accordingly, the SNPs of TLRs might change the aforementioned pathways, resulting in cancer proneness. These findings can contribute to the development of targeted preventive strategies and personalized therapeutic interventions for individuals at high risk of GC.

Our systematic review and meta-analysis provided a comprehensive evaluation of the current evidence for the association of TLR SNPs to GC risk. To the best of our knowledge, this meta-analysis was the largest review to date exploring the association between TLR-2-196 to -174de (Delta22), TLR-4 rs11536889, TLR-4 rs4986790, and TLR-4 rs4986791 polymorphisms and GC risk. This study was also the first meta-analysis on the relation of TLR-1 rs4833095, TLR-2 rs3804100, TLR-2 rs3804099, TLR-4 rs1927914, TLR-4 rs11536878, TLR-4 rs2770150, TLR-4 rs10116253, TLR-4 rs1927911, TLR-4 rs10983755, TLR-4 rs10759932, TLR-5 rs5744174, TLR-9 rs5743836, TLR-9 rs187084, and TLR-10 rs10004195 SNPs to GC susceptibility.

As a strength, no evidence of publication bias was detected, and studies that deviated from the HWE were excluded from the analyses. Nevertheless, some limitations of the present meta-analysis should be considered. First, there was significant heterogeneity across the analyzed publications. To improve the reliability of the results, the random-effects model was used for analyses. Moreover, stratified analysis revealed that ethnicity, the source of controls, and the age of participants were the sources of the observed heterogeneity. The association of the TLR polymorphisms with GC was modified by ethnicity, the source of controls, and the age of participants, indicating that these factors have contributed to the differences in the results of the previous studies. Therefore, future studies should consider these factors when investigating the association between TLR polymorphisms and gastric cancer. Second, the interactions between gene–gene and gene–environmental factors, including diet, alcohol intake, smoking, and physical activity, potentially affect GC carcinogenesis; due to the unavailability of data, we could not consider interaction analyses in this study. Third, for some SNPs, a small number of studies were included, and the findings of some subgroups may be at risk of bias because of relatively low statistical power to detect associations. Therefore, findings from the stratified analyses should be interpreted cautiously and need additional validation. We also could not perform subgroup analysis by the sex of subjects because of the lack of sex-specific data in original studies. Moreover, data for the effects of the haplotype analyses and gene–environmental factor interactions on GC were not reported sufficiently in the included original studies; thus, we could not report haplotype analyses and gene–environment interactions. Lastly, no sufficient information was available for African populations, and our result, therefore, may not be expandable to these populations. Thus, further research is required to assess the impact of these SNPs on GC in various ethnicities, particularly those of African ethnicities.

## Conclusion

In conclusion, this meta-analysis revealed that variations in TLR-4, TLR-5, and TLR-9 genes were potential risk factors of GC. Additional well-designed, large-scale studies, with more detailed information concerning gene–gene and gene–environmental interactions, should be conducted on different races, especially for less investigated SNPs, to yield a better understanding of the relationship between TLR SNPs and GC risk.

## Data Availability

The original contributions presented in the study are included in the article/Supplementary Material; further inquiries can be directed to the corresponding author.
