# Supplementary material for: Common variants in toll-like receptor family genes and risk of gastric cancer: a systematic review and meta-analysis
Source: Front Genet. 2023 Nov 28;14:1280051. doi: 10.3389/fgene.2023.1280051 (PMC10715274; doi:10.3389/fgene.2023.1280051)
Supplement: Supplementary file 1 [file Table1.DOCX]

Supplementary File 1. Characteristics of studies included in systematic review

|  |  |  |  |  |  |  | |  | | **Genotype frequency** | | | | | |
| --- | --- | --- | --- | --- | --- | --- | --- | --- | --- | --- | --- | --- | --- | --- | --- |
|  |  |  |  | **Population characteristics** | | | | | **GC cases Controls** | | | | | | |
| **Author** | **Year** | **Country** | **Ethnicity** | **Sex** | **Cases** | **Controls** | **Case age** | **Control age** | **Polymorphism** | **AA** | **Aa** | **aa** | **AA** | **Aa** | **aa** |
| Yang | 2012 | Germany | Caucasian | Both | 142 | 142 | 65.6 ± 12.9 | 61.0 ± 11.1 | TLR1 rs5743681 | 7 | 56 | 79 | 11 | 35 | 96 |
| Kim | 2013 | Korea | Asian | Both | 459 | 487 | 54.8± 8.4 | 54.3± 7.4 | TLR2 rs1898830 | 124 | 228 | 107 | 135 | 255 | 97 |
| Zeng | 2011 | China | Asian | Both | 248 | 496 | 57.8 | 57.8 | TLR5 - 889T>C | 206 | 40 | 2 | 395 | 93 | 8 |
|  |  |  |  |  |  |  |  |  | TLR5 rs2072493 | 139 | 95 | 14 | 269 | 193 | 34 |
|  |  |  |  |  |  |  |  |  | TLR2 -688G>T | 233 | 14 | 1 | 463 | 33 | 0 |
| Gonzalez‑Hormazabal | 2021 | Chile | Caucasian | Both | 299 | 301 | 25 to 93) | 18 to 82) | TLR2 rs7656411 | 16 | 99 | 184 | 23 | 110 | 168 |
|  |  |  |  |  |  |  |  |  | TLR4 rs1554973 | 9 | 77 | 213 | 7 | 79 | 215 |
|  |  |  |  |  |  |  |  |  | TLR4 rs7037117 | 15 | 122 | 162 | 20 | 114 | 167 |
|  |  |  |  |  |  |  |  |  | TLR4 rs913930 | 31 | 128 | 140 | 29 | 147 | 125 |
|  |  |  |  |  |  |  |  |  | TLR5 rs75977922 | 25 | 130 | 144 | 45 | 133 | 123 |
| Schmidt | 2011 | China | Asian | Both | 60 | 162 | NR | NR | TLR4 rs16906079 | 59 | 0 | 1 | 160 | 2 | 0 |
|  |  |  |  |  |  |  |  |  | TLR4 rs16906079 | 59 | 0 | 1 | 160 | 2 | 0 |
|  |  |  |  |  |  |  |  |  | TLR5 rs5744168 | 56 | 4 | 0 | 152 | 10 | 0 |
|  |  |  |  |  |  |  |  |  | TLR2 rs5743708 | 60 | 0 | 0 | 160 | 0 | 2 |
| Li | 2020 | China | Asian | Both | 471 | 461 | NR | NR | TLR3 rs5743303 | 334 | 120 | 17 | 334 | 114 | 13 |
|  |  |  |  |  |  |  |  |  | TLR4 rs1927914 | 173 | 233 | 65 | 149 | 241 | 81 |
|  |  |  |  |  |  |  |  |  | TLR4 rs7873784 | 459 | 12^∑^ |  | 409 | 62^∑^ |  |
|  |  |  |  |  |  |  |  |  | TLR4 rs7869402 | 430 | 41^∑^ |  | 407 | 64^∑^ |  |
|  |  |  |  |  |  |  |  |  | TLR5 rs1640816 | 384 | 87^∑^ |  | 377 | 94^∑^ |  |
|  |  |  |  |  |  |  |  |  | TLR7 rs3853839 | 87 | 44 | 8 | 102 | 30 | 18 |
| Liu | 2015 | China | Asian | Both | 209 | 94 | 59.2 ± 11.2 | 55.7 ± 17.3 | TLR3 1377C T (rs3775290) | 75 | 84 | 50 | 53 | 30 | 11 |
| Castano-Rodriguez | 2014 | China | Asian | Both | 85 | 213 | 65.3 ± 13.2 | 54.3 ± 12.8 | TLR4 rs10759931 | 42 | 32 | 11 | 78 | 106 | 29 |
|  |  |  |  |  |  |  |  |  | TLR4 rs11536891 | 70 | 14 | 1 | 172 | 39 | 2 |
|  |  |  |  |  |  |  |  |  | TLR4 rs11536898 | 70 | 14 | 1 | 168 | 40 | 2 |
|  |  |  |  |  |  |  |  |  | TLR4 rs2149356 | 42 | 32 | 11 | 80 | 103 | 29 |
|  |  |  |  |  |  |  |  |  | TLR4 rs5030728 | 85 | 0 | 0 | 210 | 3 | 0 |
| Companioni | 2014 | Some European countries | Caucasian | Both | 365 | 1281 | 58.4 + 7.93 | 58.4 + 7.69 | TLR4 rs1329061 | 150 | 166 | 49 | 572 | 580 | 129 |
|  |  |  |  |  |  |  |  |  | TLR4 rs1329060 | 262 | 91 | 12 | 948 | 309 | 26 |
|  |  |  |  |  |  |  |  |  | TLR4 rs1329057 | 222 | 126 | 17 | 825 | 410 | 48 |
|  |  |  |  |  |  |  |  |  | TLR4 rs10491851 | 217 | 126 | 21 | 678 | 516 | 87 |
| Wang | 2018 | China | Asian | Both | 312 | 380 | 49.75 ±6.71 | 51.42 ± 5.48 | TLR4 Rs1057317 | 109 | 140 | 63 | 160 | 167 | 53 |
| Xu | 2017 | China | Asian | Both | 1300 | 1300 | 59.99 ± 10.56 | 59.35 ± 9.76 | TLR5 rs5744140 | 1069 | 212 | 19 | 1098 | 189 | 13 |
|  |  |  |  |  |  |  |  |  | TLR5 rs5744113 | 768 | 440 | 92 | 772 | 448 | 80 |
|  |  |  |  |  |  |  |  |  | TLR5 rs1640827 | 842 | 406 | 52 | 901 | 366 | 33 |
|  |  |  |  |  |  |  |  |  | TLR5 rs2241096 | 762 | 471 | 67 | 772 | 456 | 52 |
|  |  |  |  |  |  |  |  |  | TLR5 rs17163737 | 679 | 509 | 112 | 745 | 468 | 87 |
|  |  |  |  |  |  |  |  |  | TLR5 rs2241097 | 974 | 307 | 19 | 990 | 296 | 14 |
| De Re | 2019 | Italy | Caucasian | Both | 43 | 52 | 61.45 ± 1.04 | 54.59 ± 1.79 | TLR8 rs3764880 | 19 | 21 | 3 | 30 | 18 | 4 |
| Gao | 2020 | China | Asian | Both | 282 | 274 | 59.48±11.23 | 59.10±11.57 | TLR9 rs164640 | 95 | 134 | 53 | 93 | 138 | 43 |

NR, not reported; ^∑^data refer to (Aa + aa).
