# Supplementary material for: Common variants in toll-like receptor family genes and risk of gastric cancer: a systematic review and meta-analysis
Source: Front Genet. 2023 Nov 28;14:1280051. doi: 10.3389/fgene.2023.1280051 (PMC10715274; doi:10.3389/fgene.2023.1280051)

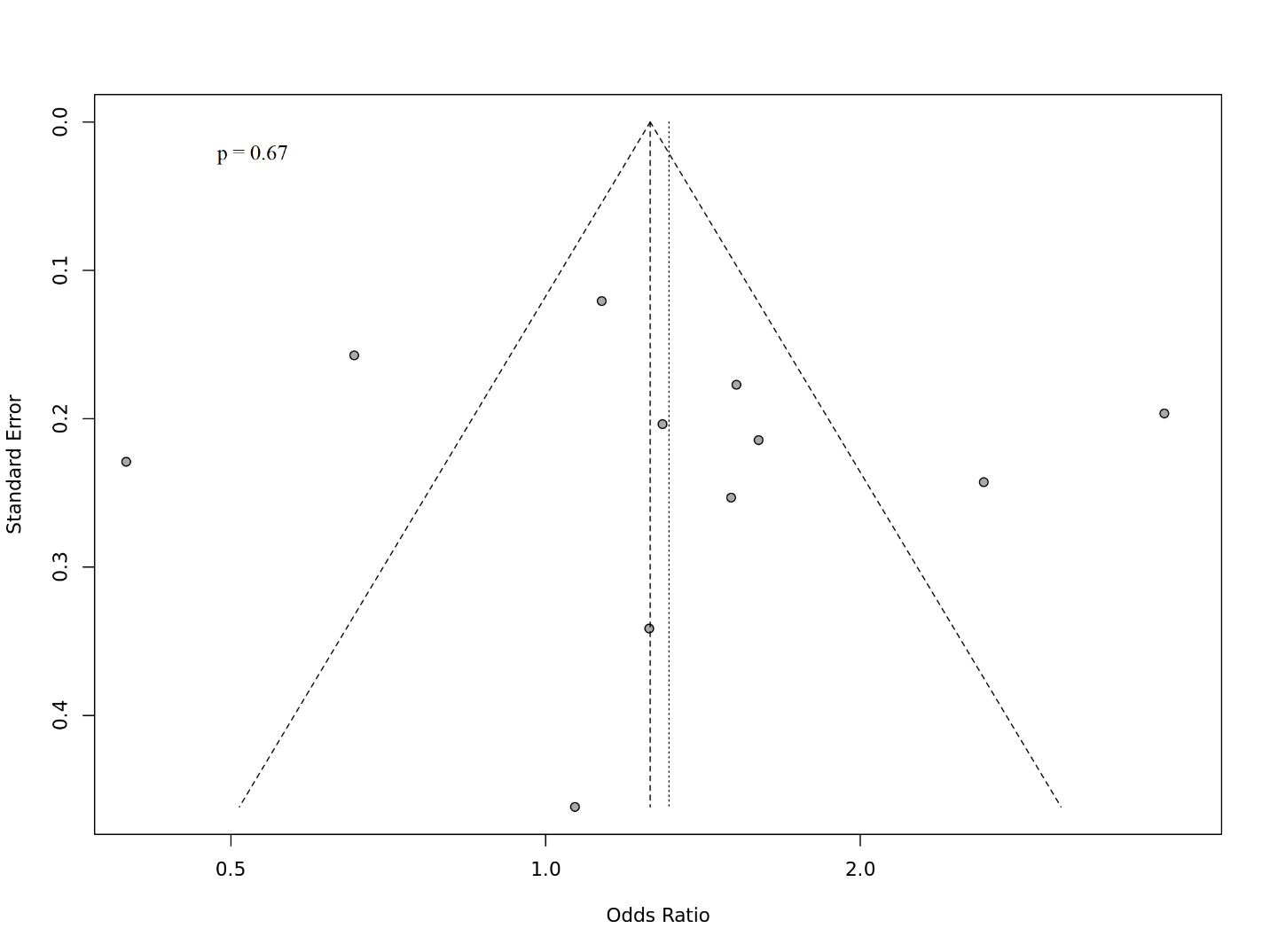


Fig S1. Funnel plot for publication bias in the studies on the association of TLR-2 -196 to - 174de (Delta22) with gastric cancer under the dominant model.

Fig S2. Funnel plot for publication bias in the studies on the association of TLR-4 rs4986790 with gastric cancer under the dominant model.


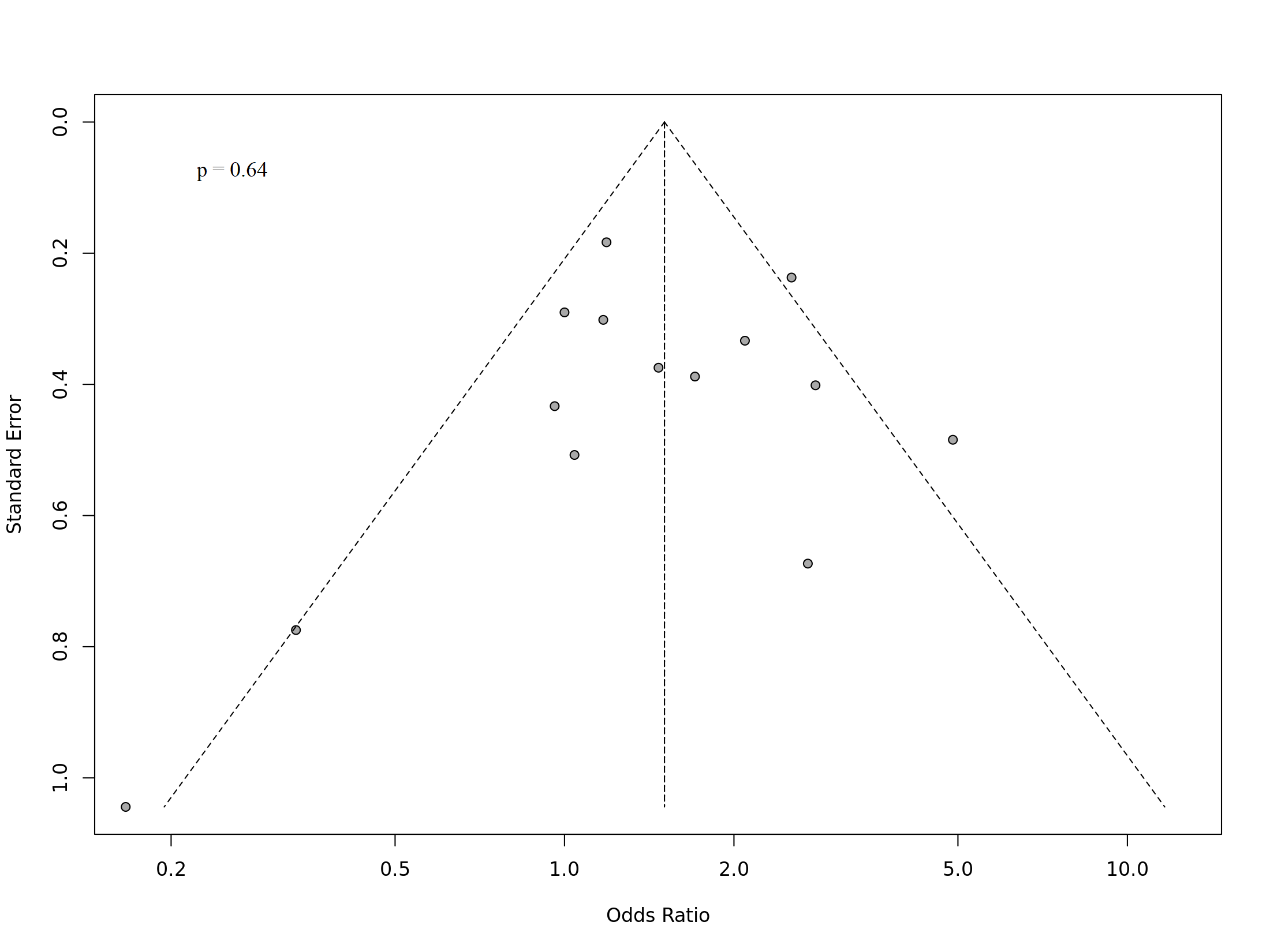


Fig S3. Funnel plot for publication bias in the studies on the association of TLR-4 rs4986791 with gastric cancer under the dominant model.


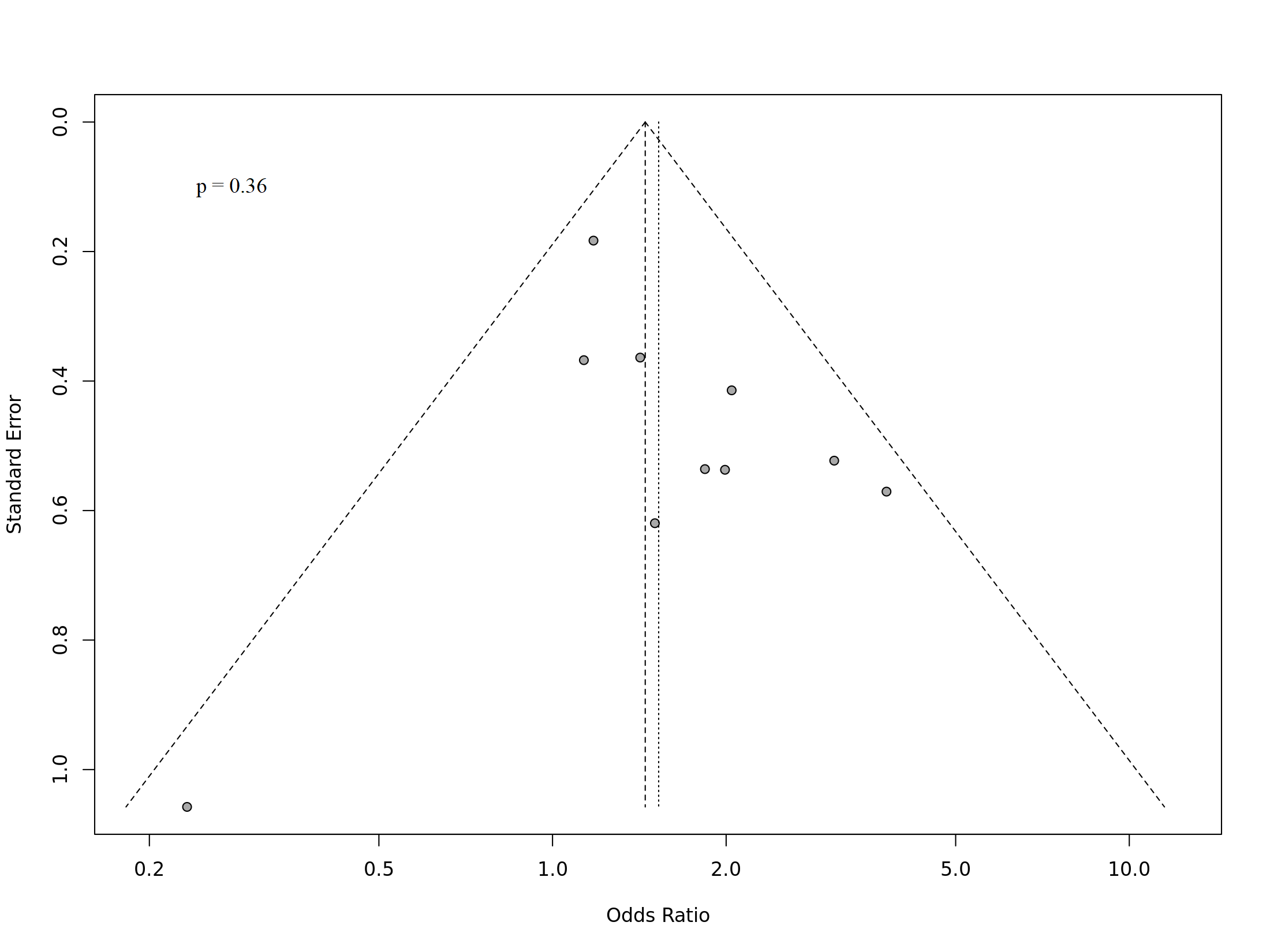

Supplement: Supplementary file 2 [file Table2.DOCX]
